# DSM265 for *Plasmodium falciparum* chemoprophylaxis: a randomised, double blinded, phase 1 trial with controlled human malaria infection

**DOI:** 10.1016/S1473-3099(17)30139-1

**Published:** 2017-06

**Authors:** Mihály Sulyok, Thomas Rückle, Alexandra Roth, Raymund E Mürbeth, Stephan Chalon, Nicola Kerr, Sonia Schnieper Samec, Nathalie Gobeau, Carlos Lamsfus Calle, Javier Ibáñez, Zita Sulyok, Jana Held, Tamirat Gebru, Patricia Granados, Sina Brückner, Christian Nguetse, Juliana Mengue, Albert Lalremruata, B Kim Lee Sim, Stephen L Hoffman, Jörg J Möhrle, Peter G Kremsner, Benjamin Mordmüller

**Affiliations:** a Institute of Tropical Medicine, and German Center for Infection Research, partner site Tübingen, Eberhard Karls University, Tübingen, Germany; b Medicines for Malaria Venture, Geneva, Switzerland; c Sanaria Inc, Rockville, MD, USA

## Abstract

**Background:**

A drug for causal (ie, pre-erythrocytic) prophylaxis of *Plasmodium falciparum* malaria with prolonged activity would substantially advance malaria control. DSM265 is an experimental antimalarial that selectively inhibits the parasite dihydroorotate dehydrogenase. DSM265 shows in vitro activity against liver and blood stages of *P falciparum*. We assessed the prophylactic activity of DSM265 against controlled human malaria infection (CHMI).

**Methods:**

At the Institute of Tropical Medicine, Eberhard Karls University (Tübingen, Germany), healthy, malaria-naive adults were allocated to receive 400 mg DSM265 or placebo either 1 day (cohort 1A) or 7 days (cohort 2) before CHMI by direct venous inoculation (DVI) of 3200 aseptic, purified, cryopreserved *P falciparum* sporozoites (PfSPZ Challenge; Sanaria Inc, Rockville, MD, USA). An additional group received daily atovaquone-proguanil (250-100 mg) for 9 days, starting 1 day before CHMI (cohort 1B). Allocation to DSM265, atovaquone-proguanil, or placebo was randomised by an interactive web response system. Allocation to cohort 1A and 1B was open-label, within cohorts 1A and 2, allocation to DSM265 and placebo was double-blinded. All treatments were given orally. Volunteers were treated with an antimalarial on day 28, or when parasitaemic, as detected by thick blood smear (TBS) microscopy. The primary efficacy endpoint was time-to-parasitaemia, assessed by TBS. All participants receiving at least one dose of chemoprophylaxis or placebo were considered for safety, those receiving PfSPZ Challenge for efficacy analyses. Log-rank test was used to compare time-to-parasitemia between interventions. The trial was registered with ClinicalTrials.gov, number NCT02450578.

**Findings:**

22 participants were enrolled between Oct 23, 2015, and Jan 18, 2016. Five participants received 400 mg DSM265 and two participants received placebo 1 day before CHMI (cohort 1A), six participants received daily atovaquone-proguanil 1 day before CHMI (cohort 1B), and six participants received 400 mg DSM265 and two participants received placebo 7 days before CHMI (cohort 2). Five of five participants receiving DSM265 1 day before CHMI and six of six in the atovaquone-proguanil cohort were protected, whereas placebo recipients (two of two) developed malaria on days 11 and 14. When given 7 days before CHMI, three of six volunteers receiving DSM265 became TBS positive on days 11, 13, and 24. The remaining three DSM265-treated, TBS-negative participants of cohort 2 developed transient submicroscopic parasitaemia. Both participants receiving placebo 7 days before CHMI became TBS positive on day 11. The only possible DSM265-related adverse event was a moderate transient elevation in serum bilirubin in one participant.

**Interpretation:**

A single dose of 400 mg DSM265 was well tolerated and had causal prophylactic activity when given 1 day before CHMI. Future trials are needed to investigate further the use of DSM265 for the prophylaxis of malaria.

**Funding:**

Global Health Innovative Technology Fund, Wellcome Trust, Bill & Melinda Gates Foundation through Medicines for Malaria Venture, and the German Center for Infection Research.

## Introduction

Despite all the success achieved in reducing the burden of malaria, in 2015 nearly half a million people died from the disease.^1^ No malaria vaccine is widely available, and chemoprophylaxis is the most efficacious method to prevent infection.

More than 100 million travellers from non-tropical regions visit malaria-endemic countries every year, of which an estimated 30 million travellers are at risk of developing malaria, and about 30 000 acquire the disease.^2^, ^3^ Both mefloquine and atovaquone-proguanil are routinely used by travellers for chemoprophylaxis. Mefloquine, given weekly, is associated with side-effects; in particular, it can cause substantial psychiatric disturbances, which have been widely discussed by regulatory authorities.^4^ Atovaquone-proguanil is well tolerated, but requires daily dosing. Non-compliance with the regimen is the most important risk factor for the failure of malaria chemoprophylaxis.^5^ Drugs for prophylaxis must be particularly efficacious, safe, and well tolerated since they are given to healthy people without substantial immunity against malaria.

**Research in context**

**Evidence before this study**

DSM265 is a selective inhibitor of the plasmodial dihydroorotate dehydrogenase. On Jan 30, 2017, we did an unrestricted PubMed database search with the term “DSM265” and found nine publications: four reviews and five original articles, none of which were a clinical trial. One study reported the preclinical evaluation of DSM265. We searched the ClinicalTrials.gov database on the same day with the same term and found six clinical trial registrations: two in Australia (first -in-man study and induced blood stage malaria challenge studies), two in the USA, one in Peru, and the reported trial in Germany. None of these studies have yet reported results.

**Added value of this study**

To our knowledge, this study is the first to report use of DSM265 for prophylaxis of malaria in human beings. Besides safety, tolerability, and pharmacokinetics of a single dose of 400 mg DSM265, protective efficacy was assessed using controlled human malaria infection (CHMI). To our knowledge, it is also the first time that a chemoprotective regimen was assessed using the highly standardised CHMI model with purified, cryopreserved *Plasmodium falciparum* sporozoites (PfSPZ Challenge).

**Implications of all the available evidence**

Our study shows that a single dose of 400 mg DSM265 is a well tolerated and efficacious antimalarial for causal prophylaxis of *P falciparum* malaria when given 1 day before direct venous inoculation of PfSPZ Challenge. Efficacy was lower when DSM265 was given 7 days before direct venous inoculation of PfSPZ Challenge but partial activity against the asexual blood stage was maintained. Future trials are needed to optimise DSM265 formulation, dose, and schedule, and assess its activity against naturally acquired *P falciparum* malaria.

The target candidate and product profiles for antimalarials were recently updated with particular emphasis on elimination and eradication scenarios,^6^ which requires candidates with chemoprophylactic activity, a drug class with a comparatively dry pipeline until recently.^7^ One compound, tafenoquine, has been in late-stage clinical development for more than 15 years.^8^ The current pipeline of antimalarial drugs that are in preclinical development looks more promising. Compounds with potential for prophylactic use (ie, DSM265, MMV390048, DDD107498, P218, DSM421, AN13762, and UCT943) are in the preclinical and early phase of clinical testing.^9^ Clinical data for these candidates are, therefore, desperately needed.

The development of new clinical study designs that allow the evaluation of chemoprophylactic interventions, including efficacy early in clinical development are of paramount importance. Applying such an improved design, the selection of the most promising drug candidate for further development in large, resource intensive licensure programmes would be more stringent and fast. The recent development of controlled human malaria infection (CHMI) models using aseptic, purified, cryopreserved *Plasmodium falciparum* sporozoites (PfSPZ Challenge) facilitates studies with the required level of standardisation and flexibility for exploratory early phase clinical trials.^10^

DSM265 is the most advanced of a new generation of antimalarial drugs with the potential to prevent infection with *P falciparum* parasites. It is a novel, orally active plasmodium-selective dihydroorotate dehydrogenase (DHODH) inhibitor^11^ with a long elimination half-life in animal models.^12^ DHODH is a key enzyme of pyrimidine biosynthesis, and *P falciparum*, unlike most human tissues, lacks salvage pathways that could serve as an alternative source of pyrimidines.^11^ DSM265 acts in vitro against both the pre-erythrocytic *P falciparum* life-cycle stage, and against asexual blood stages.^12^ However, it has only modest activity against sexual life-cycle stages of plasmodia.^12^ DSM265 is a particularly promising candidate for clinical development for chemoprophylaxis since it might have causal (ie, pre-erythrocytic) prophylactic activity (chemoprotection), and a pharmacokinetic profile that potentially supports weekly administration. Besides this study, five other yet-unpublished clinical trials on DSM265 are close to completion, with a focus on safety, tolerability, bioavailability, and efficacy against uncomplicated *P falciparum* and *Plasmodium vivax* malaria (NCT02389348, NCT02573857, NCT02123290, NCT02562872, and NCT02750384).

The aim of our study was to assess the safety, tolerability, and pharmacokinetics of DSM265 as well as the chemoprophylactic efficacy of DSM265 by CHMI, using direct venous inoculation (DVI) of 3200 *P falciparum* sporozoites (PfSPZ Challenge).

## Methods

### Study design and participants

**Figure 1:**
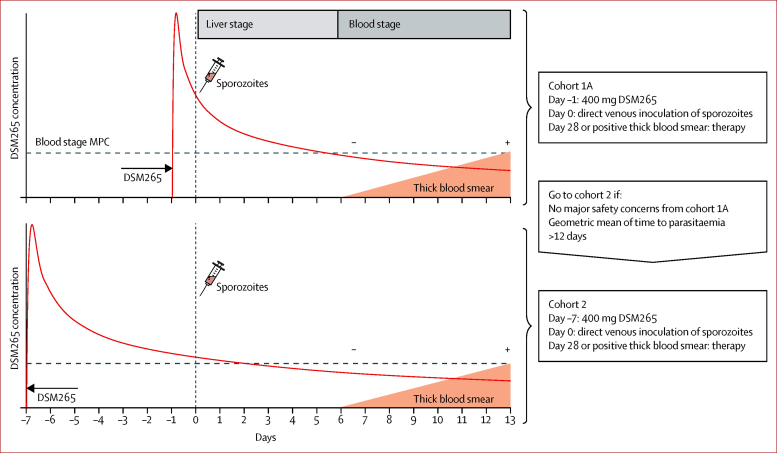
Study design and main interventions Red curves show the expected DSM265 concentrations. MPC=minimal parasiticidal concentration.

This single centre, double-blind, randomised, placebo-controlled phase 1 clinical trial recruited two sequential cohorts (figure 1): cohort 1A had eight volunteers receiving a single dose of either 400 mg DSM265 (n=6) or placebo (n=2) 1 day before CHMI (day −1); cohort 2 had eight volunteers receiving a single dose of either 400 mg DSM265 (n=6) or placebo (n=2) 7 days before CHMI (day −7). In parallel to cohort 1A, six volunteers (cohort 1B) received atovaquone-proguanil (250-100 mg) daily for 9 days, starting 1 day before DVI (day −1). The study of cohort 1B was open label. All volunteers underwent CHMI by DVI of 3200 *P falciparum* sporozoites by PfSPZ Challenge. The day of DVI for CHMI was defined as day 0 for all study procedures (eg, DSM265 dosing 7 days before DVI [day −7]).

Recruitment for cohort 2 depended on at least partial efficacy of DSM265 in cohort 1A (geometric mean time-to-parasitaemia, assessed by thick blood smear [TBS] after 12 days), and on a positive safety review by a safety review team. The last scheduled follow-up visit was on day 60 following DVI for CHMI (day 60).

The study population was selected to represent healthy, malaria-naive adults. Volunteers aged 18–45 years with a body-mass index (BMI) between 18 kg/m^2^ and 30 kg/m^2^ were included. All participants were required to practice effective contraception, and female participants were to provide a negative pregnancy test. Further inclusion criteria were: no clinically significant findings in history, physical, and laboratory examination, being reachable at all times by mobile phone during the whole study, agreement to share medical information on the volunteer with his or her general practitioner, and understanding of study procedures and risks, assessed by a multiple choice test. Additionally, willingness to undergo CHMI with PfSPZ Challenge, to take a curative regimen if necessary, and being able to comply with all study requirements (in the investigator's opinion) were also required.

Exclusion criteria were: a history of malaria or plans to travel to endemic regions during the study, participation in another clinical trial within 30 days before enrolment or during the study, previous participation in a malaria vaccine trial, history of serious psychiatric conditions, convulsions, or severe head trauma, any malignancy, and diabetes mellitus. Moreover, arrhythmias, prolonged QTc interval (>450 ms), or any other clinically significant abnormalities in the electrocardiogram, breast feeding, or intention to become pregnant, HIV, hepatitis B or C virus infection, any suspected immunodeficient state, history of splenectomy, and haemoglobinopathies also prevented participation. Further exclusion criteria were smoking more than ten cigarettes or equivalent per day, alcohol or injected drug abuse, moderate risk or higher categories for cardiovascular events within 5 years of the study commencement date, use of any prescription drugs (except contraception), herbal supplements, or over-the-counter medication within 2 weeks before initial dosing, and intake of grapefruit, grapefruit juice, Seville orange, or other products containing these ingredients within 7 days of the first drug administration of DSM265 or placebo. Any symptoms, physical signs, and laboratory values suggestive of systemic disorders, or of conditions that could interfere with the interpretation of the study results, or compromise the health of the volunteers excluded participation. The full study protocol with further details is available from the corresponding author upon request. Eligibility criteria were assessed after written informed consent was given.

The study was done at the clinical trial platform of the Institute for Tropical Medicine in Tübingen, Germany, and received approval from the ethics committee of the Eberhard Karls University and the University Clinics Tübingen. The study was compliant with the International Council for Harmonisation Good Clinical Practice guidelines and the German Medicines Law.

### Randomisation and masking

Randomisation for cohort 1 and cohort 2 was done by an interactive web response system service provided by an external contract research organisation, which attributed a volunteer identification code to each eligible volunteer using a computer-generated (SAS v9.2, Proc Plan procedure) allocation sequence without restrictions. In cohort 1, allocation to cohort 1A (DSM265 and placebo) or 1B (atovaquone-proguanil) was open label. Nested in cohort 1A, allocation to DSM265 or placebo was double-blinded as it was in cohort 2. Clinical team, funder, and volunteers remained blinded until database lock. Only the study pharmacist received the randomisation list in a sealed envelope on the day of DSM265 administration and had no other role in the trial. The allocation ratio for DSM265 to placebo was 3:1. A higher proportion of DSM265 against placebo recipients was chosen to increase information on efficacy, safety, and tolerability of DSM265. The atovaquone–proguanil group served as an additional control.

Placebo was taste-blended, and visually and haptically indistinguishable from DSM265. Following preparation by the study pharmacist, the product was transferred to the clinical department by a blinded courier. Volunteers were required to ingest the DSM265 or placebo suspension under observation of at least two investigators. All containers used were identical, and were marked with the volunteer identification code. First unblinding was done following an interim database lock after day 28 of cohort 1A to allow assessment of efficacy by an independent statistician. The sponsor and investigators remained blinded until completion of follow-up (day 60). Cohort 2 was unblinded after final database lock.

### Procedures

DSM265 (Bend Research Inc, Bend, OR, USA) was supplied as a 25% (250 mg/g) spray-dried dispersion. The powder was reconstituted in 240 mL of vehicle (0·1% methocel A4M, 0·1% polysorbate 80, 0·005% simethicone emulsion, 0·5% sucralose, and 0·05% ethyl vanillin) on day of dosing. The placebo (Bend Research Inc, Bend, OR, USA) was supplied as hydroxypropylmethylcellulose acetate succinate powder in a bottle and reconstituted exactly as DSM265. DSM265 and placebo were given orally as liquid emulsion.

Volunteers from cohort 1A and cohort 2 were dosed after fasting for at least 4 h, followed by a standardised breakfast after drug intake. Meal records were taken until day 28 of CHMI. In cohort 1B, atovaquone-proguanil (Malarone, GlaxoSmithKline, München, Germany) was given daily for 9 days together with a fat-containing snack or drink, as recommended on the package insert. Blood for pharmacokinetic analyses was sampled at baseline, 15 min, 30 min, 60 min, and 2 h, 4 h, 6 h, 8 h, 14 h, 24 h, 48 h, 72 h, 168 h, 264 h, and 480 h after dosing in cohort 1A and cohort 2 for pharmacokinetic analyses. DSM265 and DSM450 (the main metabolite) concentrations in blood were measured from plasma and dried blood spots by liquid chromatography-tandem mass spectrometry at Swiss BioQuant (Reinach, Switzerland), according to standard operating procedures (details available upon request from the corresponding author).

CHMI was initiated by DVI of 3200 purified, cryopreserved *P falciparum* sporozoites, strain NF54 (PfSPZ Challenge; Sanaria Inc, Rockville, MD, USA) as previously described.^10^, ^13^ All participants received PfSPZ Challenge from the same lot. From day 6 following DVI, parasitaemia was monitored daily by TBS and quantitative PCR (qPCR) until TBS positivity or day 28. TBS procedures allowed detection of at least ten parasites per μL with greater than 95% probability.^14^ qPCR was done using published procedures with minor modifications;^15^ 0·5 mL of fresh blood was extracted using silica-coated magnetic particles (EZ1 DNA blood; Qiagen, Hilden, Germany) and amplified in 384-well plates using a LightCycler 480 (Roche, Mannheim, Germany). Lower limit of detection of qPCR was three parasites per mL. DVI for CHMI was done either 1 day (cohort 1) or 7 days (cohort 2) after the first dose of chemoprophylaxis. Volunteers who became TBS positive were treated with artemether-lumefantrine, atovaquone-proguanil, or chloroquine according to the manufacturer's specifications. All other volunteers received a treatment course on day 28. All treatments and injections were given by one and verified by another investigator.

Adverse events and concomitant medications were captured during all on-site visits and evaluated by the investigators. From day 6 following DVI onwards, blood for parasitological investigations was sampled daily until day 28. In volunteers who became TBS positive, daily sampling was continued until two consecutive TBS were negative. Additionally, volunteers were contacted by telephone at prespecified intervals. The clinical team was available at all times for the duration of the trial. Biochemistry, haematology, and urine analysis were scheduled regularly (screening, day of treatment, day of malaria, days 0, 1, 7, 14, 21, 28, and 60 of CHMI), and a 12-lead electrocardiogram (ECG) was done before, at 8 h and 24 h after dosing, before malaria treatment, and on day 28 and day 60 of CHMI.

### Outcomes

The primary efficacy endpoint was the prepatent period in days defined as time from DVI of PfSPZ Challenge (day 0) to parasitaemia, detected by TBS. Secondary outcome measures were number and severity of adverse events, the pharmacokinetic (DSM265 and DSM450 concentration in peripheral blood over time), and pharmacodynamics profile (parasitaemia over time).

### Statistical analysis

The sample size of this study reflects the exploratory nature of the trial. Sample size was selected to demonstrate at least 61% protective efficacy with 95% probability when full protection (six of six) is achieved (exact one-sided 95% CI 0·61–1). Infectivity of the inoculum (3200 *P falciparum* sporozoites of PfSPZ Challenge) in malaria-naive volunteers is 100% with 56 of 56 exposed volunteers enrolled in independent studies at the time of writing the protocol (exact one-sided 95% CI 0·95–1).^10^, ^13^, ^16^, ^17^ All participants receiving at least one dose of chemoprophylaxis or placebo were considered for safety, those receiving CHMI with PfSPZ Challenge for efficacy. A non-compartmental model was used for pharmacokinetic analysis (WinNonlin v6.3, Pharsight Corporation, Mountain View, CA, USA). Concentration (C_max_) and time (t_max_) at peak levels were read from the concentration-over-time plot and area under the curve (AUC) was calculated using the linear trapezoidal rule. The safety review team reviewed safety, pharmacokinetic, and pharmacodynamics data on completion of the CHMI in each cohort (day 28) and decided on trial continuation or discontinuation, serious adverse events, or any other crucial findings. The Wilcoxon-Mann-Whitney test was used for comparisons of non-parametric variables. Log-rank test was used to compare time-to-parasitaemia between placebo and DSM265 treatments. All analyses were coded with R,^18^ and SAS 9.2 (SAS Institute, Cary, NC, USA). The study was registered with ClinicalTrials.gov, number NCT02450578.

### Role of the funding source

The study was funded by the Global Health Innovative Technology Fund, Wellcome Trust, and Bill & Melinda Gates Foundation through Medicines for Malaria Venture, and by the German Center for Infection Research. TR, JJM, NG, and SC are employees of Medicines for Malaria Venture who contributed to study design and data interpretation. Medicines for Malaria Venture had no role in data collection. Other funders had no role in study design, data collection, data analysis, data interpretation, or writing of the report. The authors had full access to all the data in the study, and share responsibility for the decision to submit for publication.

## Results

**Figure 2:**
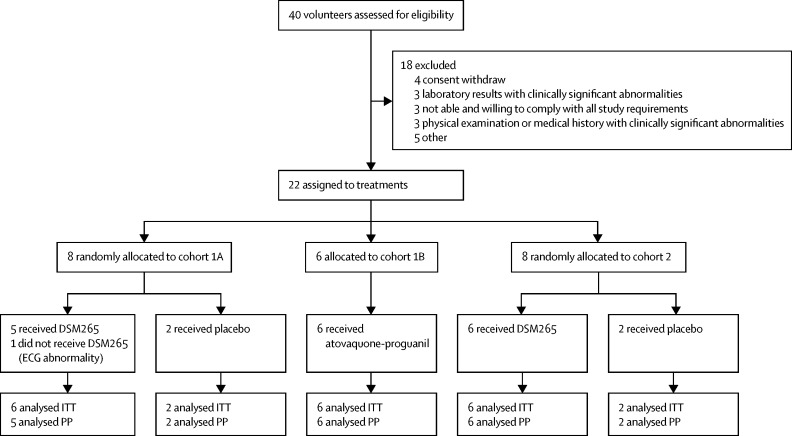
Trial profile ECG=electrocardiogram. ITT=intention to treat. PP=per protocol.

Participants were recruited between Oct 23 and Nov 13, 2015, for cohorts 1A and 1B, and between Jan 12 and Jan, 28, 2016, for cohort 2. 22 (55%) of 40 screened volunteers were eligible. Volunteers were allocated to receive placebo, atovaquone-proguanil, or DSM265, in cohort 1, and placebo or DSM265 in cohort 2 (figure 2). One participant in cohort 1A did not receive DSM265 because of transient second-degree atrioventricular-block detected on ECG immediately before dosing. 14 (64%) of the 22 randomly assigned volunteers were male. Baseline demographic characteristics were similar between cohorts and treatment groups (table). No volunteer was lost to follow-up.

**Table:** Baseline characteristics of the intention-to-treat population

	**Cohort 1A**		**Cohort 1B**	**Cohort 2**	
	DSM265 (n=6)	Placebo (n=2)	Atovaquone-proguanil (n=6)	DSM265 (n=6)	Placebo (n=2)
Age (years)	25·0 (22·0–28·0)	27·0 (25·0–29·0)	25·5 (23·5–29·8)	25·5 (22·3–28·8)	25·0 (23·5–26·5)
Sex (male)	4 (66%)	1 (50%)	4 (66%)	4 (66%)	1 (50%)
Weight (kg)	67·5 (58·3–79·0)	70·0 (59·5–80·5)	76·0 (64·8–91·0)	87·0 (72·8–88·5)	64·0 (63·5–64·5)
Height (cm)	178·0 (167·5–179·5)	165·0 (160·0–170·0)	181·0 (168·8–182·0)	181·5 (177·8–185·2)	167·5 (163·8–171·2)
Body-mass index (kg/m^2^)	22·3 (20·4–23·9)	25·0 (22·7–27·4)	24·5 (22·4–27·9)	25·7 (24·0–26·7)	22·9 (22·0–23·8)

Data are n (%) and median (IQR).

In cohort 1 all volunteers treated with DSM265 and atovaquone-proguanil were fully protected; no parasitaemia in TBS and qPCR occurred during follow-up. All volunteers (n=4) allocated to placebo in cohort 1A and cohort 2 developed parasitaemia with a geometric mean time-to-parasitaemia by TBS microscopy (prepatent period) of 11·7 days (SD 1·1; cohort 1A: 11 and 14 days; cohort 2: 11 and 11 days). The geometric mean of time-to-parasitaemia by qPCR was 7·8 days (SD 1·3; days 7, 7, 7, and 11).

**Figure 3:**
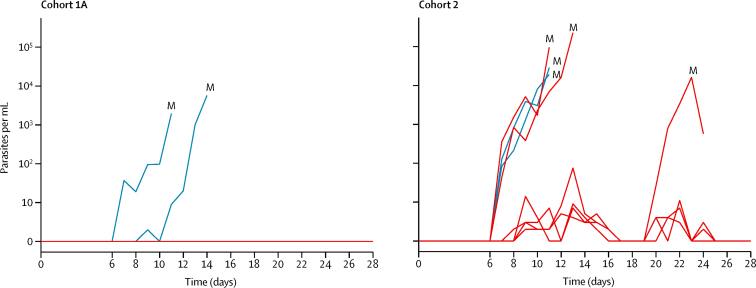
Parasitaemia assessed by quantitative PCR Placebo volunteers (blue) and DSM265 volunteers (red). In cohort 1A, all DSM265 volunteers remained negative, in cohort 2 all became positive. M=malaria defined as positive thick blood smear.

Cohort 2 volunteers were partly protected (figure 3): three of six DSM265-treated volunteers developed parasitaemia detected by TBS, with a geometric mean prepatent period of 15·1 days (SD 1·5; days 11, 13, and 24). The prepatent period in DSM265-treated volunteers of cohort 2 compared with placebo recipients was longer (p=0·036, log-rank test). However, all DSM265-treated volunteers of cohort 2 developed parasitaemia, detected by qPCR (figure 3). Peak parasite density in cohort 2 was a median of 17 702 parasites per mL (IQR 60–45 550) reached at a median of 11·5 days (11·0–13·0). Geometric mean time to qPCR positivity was 8·2 days (SD 1·2). The highest parasite load assessed by TBS microscopy was a median of 11 parasites per μL (IQR 4–17), and did not differ significantly between placebo and DSM265 recipients (p=0·4). In cohort 2, three of the DSM265-treated volunteers developed submicroscopic parasitaemia, did not develop malaria symptoms, and remained TBS negative between day 25 and day 28 when they received treatment. Peak parasite density in the four placebo recipients (cohort 1A: 1955 parasites per mL and 5691 parasites per mL; cohort 2: 28 756 parasites per mL and 19 208 parasites per mL) was reached at the time of TBS positivity.

All TBS-positive volunteers received a full therapeutic course of atovaquone-proguanil (1000-400 mg), once daily for 3 days, starting on the day of TBS positivity. Treatment was successful in all cases, and parasitaemic volunteers recovered without sequelae.

All seven volunteers in cohort 1A experienced adverse events: there were 41 grade 1, three grade 2, one grade 3, and two grade 4 events. The only possible DSM265-related adverse event was an episode of transient increase in total, direct, and indirect serum bilirubin 2–8 days following dosing. A similar transient elevation was observed as a baseline (pre-treatment) finding in the same participant. During follow-up, one serious adverse event occurred in cohort 1A: a bilateral pulmonary embolism occurred in a non-smoking woman under oral contraceptive treatment 34 days after DSM265 administration while she was on a transatlantic flight. The volunteer never became parasitaemic and recovered fully, except for a grade 1 sinus tachycardia, which still persisted at the last follow-up visit.

In cohort 1B, 20 grade 1 and one grade 2 adverse events occurred in five of six volunteers. Five adverse events were attributed to atovaquone-proguanil; all grade 1 in severity (headache, nausea, constipation, epigastric discomfort, and hyperhidrosis). No adverse events were attributed to malaria.

In cohort 2, 108 adverse events were reported in eight volunteers: 81 grade 1, 19 grade 2, six grade 3, and two grade 4 events. The most frequent adverse events were fatigue (16 episodes in all eight volunteers: 12 grade 1, three grade 2, and one grade 3) and headache (15 episodes in six volunteers: ten grade 1 and five grade 2), followed by fever (14 episodes in six volunteers: four grade 1, six grade 2, and four grade 3). 60 adverse events were attributed to malaria. The only two severe adverse events (grade 3 and 4) not attributed to malaria were laboratory abnormalities (hyperkalaemia) without clinical signs, most likely due to sampling error. DSM265-related adverse events were not reported in this cohort.

Altogether, 58 adverse events (48 grade 1, nine grade 2, and one grade 3) occurred in the placebo recipients in both cohorts. 29 of 58 events were associated with malaria or with antimalarial treatment. The most frequent adverse events were headache (11 episodes in four volunteers: eight grade 1 and three grade 2) followed by fatigue (nine episodes in four volunteers: seven grade 1, one grade 2, and one grade 3), and fever (six episodes in two volunteers: three grade 1 and three grade 2). Drug-related adverse events were observed after placebo administration in three of four participants: insomnia (one event in one volunteer), abdominal discomfort (two events in two volunteers), palpitations (three events in one volunteer), dizziness (two events in one volunteer), and headache (one event in one volunteer).

QTc prolongation was not observed in the DSM265-treated participants. Treatment-emergent adverse events are listed in the appendix (pp 20–38).

In pharmacokinetics analysis, the concentration of DSM265 and DSM450 sampled from plasma and dried blood spots were in agreement, although the concentration of both molecules was systematically lower in dried blood spots over the whole concentration range, probably because of the paper matrix (appendix pp 3–16). Therefore, only plasma concentrations are reported (a full analysis is given in the appendix pp 3–18).

**Figure 4:**
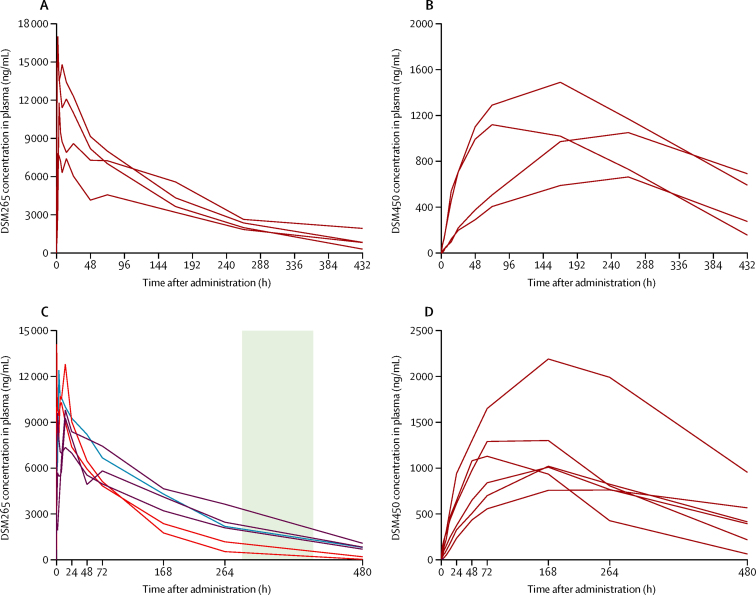
Concentration of DSM265 and DSM450 over time Individual (spaghetti) concentration–time profiles in plasma of DSM265 (A and C) and DSM450 (B and D) following administration of a single dose of 400 mg DSM265 in cohort 1 (A and B) and cohort 2 (C and D). Participants in cohort 2 (C) who developed malaria on days 11 and 13 are shown in red, the participant with malaria on day 24 is shown in blue, and participants with submicroscopic parasitaemia without malaria are shown in purple. The green shading shows the approximate time of the first cycle of asexual parasitaemia.

Median C_max_ was 12·1 μg/mL (IQR 9·6–13·8), median t_max_ was 3 h (2–12). The median plasma elimination half-life for DSM265 was 137·5 h (IQR 95·1–147·5). AUC_0–∞_ values in plasma ranged from 1080 μg·h/mL to 2460 μg·h/mL (median 1745 μg·h/mL [IQR 1442–2060]). Median AUC_0–168_ was 1031 μg·h/mL (IQR 867–1165). The pharmacokinetics of DSM265 and DSM450 showed a similar profile in cohort 1A and cohort 2 (figure 4).

Since all volunteers in cohort 2 developed parasitaemia, an exploratory analysis to assess pharmacokinetic parameters with pharmacodynamics was done. The DSM265 half-life was longer (p=0·046, Wilcoxon-Mann-Whitney test) in TBS-negative (143 h, 143 h, and 149 h in each volunteer) compared with TBS-positive infections (57 h, 90 h, and 132 h in each volunteer). The concentration of DSM265 was particularly low in two participants with similar parasite kinetics to placebo (figure 4).

## Discussion

Our study shows that a single dose of 400 mg DSM265 was well tolerated and efficacious for causal chemoprophylaxis against *P falciparum* malaria when given 1 day before PfSPZ Challenge inoculation. DSM265 is currently being developed as a drug for antimalarial treatment and chemoprophylaxis.^11^ Preclinical data suggest that DSM265 is active against pre-erythrocytic and asexual blood stages of plasmodia,^12^ and therefore could be used in chemoprotection. Such a drug would not need to be given before and after exposure to infectious mosquito bites because parasites are eliminated without a substantial time lag. In this phase 1 study, we assessed for the first time whether a single, oral dose of DSM265 is safe, well tolerated, and can prevent the development of parasitaemia in malaria-naive, healthy adults, when PfSPZ are inoculated 1–7 days after drug administration. Standardised CHMI with PfSPZ Challenge^10^, ^13^ provides a new tool to assess chemoprophylactic and therapeutic properties of new chemical entities in early, exploratory, small-scale phase 1 clinical trials. CHMI can be initiated at any time (no need to breed and infect mosquitoes). Hence, sequential and adaptive study designs can be used. Infection is highly reproducible and therefore the number of participants and infection controls can be minimised compared with natural exposure and mosquito-mediated CHMI.^10^, ^19^ Also, the fully infectious dose is small enough to allow at least three replication cycles following the hepatic stage, hence activity against asexual blood stages can be assessed as wel. The present study is the first to combine all three advantages of the approach and will guide further clinical development of DSM265 as a single agent or combination partner.

A limitation of such studies is the low sample size that misses small differences and rare safety issues. When unreplaceable dropouts occur, as in cohort 1A of this study, power further decreases. Other limitations of CHMI studies are related to their model character and the study population. CHMI is optimised to infect all inoculated volunteers, which is more stringent than in naturally acquired infection, in which only a fraction of bites of infected mosquitoes will lead to malaria.^19^ Such non-physiological inoculates are required to ascertain that the prophylactic effect of the drug is measured and not infectiveness of the inoculum. Additionally, CHMI uses only a single parasite strain or clone (most frequently NF54 or its derivative 3D7), whereas under natural conditions polyclonal infections occur. In addition to these parasitological aspects, phase 1 and CHMI trials recruit only volunteers who represent a small fraction of the target population (young and healthy adults), whereas the target population includes all age strata and vulnerable groups such as pregnant women and immunosuppressed patients. Additionally, the late TBS-positive parasitaemia in one participant in cohort 2 raises questions about the risk of possible delayed occurrence of parasitaemia after day 28 in cohort 1A. However, in the participant with delayed TBS positivity, a low-grade, qPCR-detectable parasitaemia was present from day 8. By contrast, in cohort 1A, all DSM265-treated volunteers remained negative with qPCR. Thus, a late presentation after day 28 is rather unlikely in our opinion.

DSM265 was safe and well tolerated in this study's setting, with no clinically significant changes in vital signs, ECGs, or laboratory tests following oral dosing of 400 mg DSM265. The only serious adverse event, a bilateral pulmonary embolism, was not considered to be related to the investigational product, or any other study procedures such as PfSPZ Challenge or antimalarial rescue treatment. The long intercontinental flight and concomitant intake of oral contraceptives, two multiplicative risk factors for thromboembolic events, were the likely triggers for this event.^20^, ^21^

A preliminary good safety profile makes DSM265 a highly promising candidate for further development as a chemoprophylactic antimalarial. Given 1 day before CHMI, all participants were fully protected. This action contrasts with chemoprophylaxis with several approved drugs that only act against the asexual blood stage (eg, chloroquine or mefloquine). Here, parasites must egress from the liver before they become susceptible to the drug. Other drugs such as the artemisinins have extremely short half-lives and offer no protection beyond a few hours.

When given 7 days before CHMI (cohort 2), DSM265 efficacy was reduced. Prevention of malaria depended on the half-life of DSM265 and the concentration at the moment of CHMI, whereas the pre-erythrocytic development of the parasite was not sufficiently suppressed; all volunteers developed parasitaemia. Nevertheless, in three of six participants asexual blood stage growth was suppressed. Furthermore, we observed substantial variation in the time to peak plasma concentration. Despite fasting before and standardised food intake following DSM265 administration, a food effect cannot be excluded since drinking was not restricted following DSM265 intake.

Despite the small sample size, this study provides important information on the potential of DSM265 both in chemoprotection and in malaria treatment. Our study suggests that gains in formulation, dosing, and schedule that improve consistency and duration of exposure might translate to longer protection. Since no evidence for drug-related toxicity has been observed, a further dose increase can be envisaged, which we predict could prolong protective efficacy.

To our knowledge, this is the first time that standardised CHMI using DVI of PfSPZ has been used to assess chemoprotective properties of a new chemical entity in early development. We show that DSM265 is well tolerated and has causal prophylactic activity against *P falciparum* malaria that is correlated with plasma exposure of the compound.

**This online publication has been corrected. The corrected version first appeared at thelancet.com/infection on May 23, 2017.**
