# Corrections

**DOI:** 10.1016/S1473-3099(17)30277-3

**Published:** 2017-06

*Sulyok M, Rückle T, Roth A, et al. DSM265 for* Plasmodium falciparum *chemoprophylaxis: a randomised, double blinded, phase 1 trial with controlled human malaria infection.* Lancet Infect Dis *2017; **17:** 534–42—*Author name “Kim Lee Sim” has been corrected to “B Kim Lee Sim”. This correction has been made to the online version as of May 23, 2017, and the printed version is correct.

