# Supplementary material for: DSM265 for Plasmodium falciparum chemoprophylaxis: a randomised, double blinded, phase 1 trial with controlled human malaria infection
Source: Lancet Infect Dis. 2017 Jun;17(6):636–44. doi: 10.1016/S1473-3099(17)30139-1 (PMC5446410; doi:10.1016/S1473-3099(17)30139-1)
Supplement: Supplementary appendix [file mmc1.pdf]

THE LANCET
Infectious Diseases

Supplementary appendix

This appendix formed part of the original submission and has been peer reviewed. We post it as supplied by the authors.

Supplement to: Sulyok M, Rückle T, Roth A, et al. DSM265 for *Plasmodium falciparum* chemoprophylaxis: a randomised, double blinded, phase 1 trial with controlled human malaria infection. *Lancet Infect Dis* 2017; published online March 28. http://dx.doi.org/10.1016/S1473-3099(17)30139-1.

DSM265 for *Plasmodium falciparum* chemoprophylaxis: Online Appendix

Table of Contents

Pharmacokinetic analysis 3

Methods 3

Results: Cohort 1A. 3

Table S1 Mean DSM265 DBS concentrations (ng/mL) and descriptive statistics following administration of a single dose of 400 mg DSM265. 3

Table S2 Mean DSM265 plasma concentrations (ng/mL) and descriptive statistics following administration of a single dose of 400 mg DSM265. 4

Table S3 Mean DSM450 DBS concentrations (ng/mL) and descriptive statistics following administration of a single dose of 400 mg DSM265. 4

Table S4 Mean DSM450 plasma concentrations (ng/mL) and descriptive statistics following administration of a single dose of 400 mg DSM265. 5

Figure S1 Mean DSM265 and DSM450 concentration-time profiles in both DBS and plasma following administration of a single dose of 400 mg DSM265. 6

Figure S2 Individual (spaghetti) concentration-time profiles in both DBS and plasma of DSM265 and DSM450 following administration of a single dose of 400 mg DSM265 7

Table S5 Individual and mean DSM265 DBS pharmacokinetic variables following administration of single doses of 400 mg DSM265. 8

Table S6 Individual and mean DSM265 plasma pharmacokinetic variables following administration of single doses of 400 mg DSM265. 8

Table S7 Individual and mean DSM450 DBS pharmacokinetic variables following administration of single doses of 400 mg DSM265. 9

Table S8 Individual and mean DSM450 plasma pharmacokinetic variables following administration of single doses of 400 mg DSM265. 9

Figure S3 Correlation between DBS and plasma concentrations of DSM265 and DSM450 10

Results: Cohort 2 11

Table S9. Cohort 2: Mean DSM265 DBS concentrations (ng/mL) and descriptive statistics following administration of a single dose of 400 mg DSM265 11

Table S10 Cohort 2: Mean DSM265 plasma concentrations (ng/mL) and descriptive statistics following administration of a single dose of 400 mg DSM265. 12

Table S11 Cohort 2: Mean DSM450 DBS concentrations (ng/mL) and descriptive statistics following administration of a single dose of 400 mg DSM265 12

Table S12 Cohort 2: Mean DSM450 plasma concentrations (ng/mL) and descriptive statistics following administration of a single dose of 400 mg DSM265 13

Figure S4 MMV_DSM265_14_01 Cohort 2: Mean DSM265 and DSM450 concentration-time profiles in both DBS and plasma following administration of a single dose of 400 mg DSM265 14

Figure S5 Cohort 2: Individual (spaghetti) concentration-time profiles in both DBS and plasma of DSM265 and DSM450 following administration of a single dose of 400 mg DSM265 15

Table S13 Cohort 2: Individual and mean DSM265 DBS pharmacokinetic variables following administration of single doses of 400 mg DSM265 16

Table S14 Cohort 2: Individual and mean DSM265 plasma pharmacokinetic variables following administration of single doses of 400 mg DSM265 16

Table S15 Individual and mean DSM450 DBS pharmacokinetic variables following administration of single doses of 400 mg DSM265. 17

Table S16 Cohort 2: Individual and mean DSM450 plasma pharmacokinetic variables following administration of single doses of 400 mg DSM265 17

Figure S6 MMV_DSM265_14_01 Cohort 2: Correlation between DBS and plasma concentrations of DSM265 and DSM450 18

Microscopically detectable parasitaemia in Cohort 2 18

Figure S7. Kaplan-Meyer curves of Cohort 2. 18

PK/PD relationship of Cohort 1A and 2....19

Table S17. PK/PD relationship of Cohort 1A, and 2. 19

Treatment emergent adverse events 20

From 1st treatment administration up to End of Study Visit (Day 60) 20

From treatment administration to initiation of the PfSPZ challenge 28

From PfSPZ challenge up to 1st dose of rescue medication 29

From 1st dose of rescue medication up to End of Study Visit (Day 60)....33

Pharmacokinetic analysis

Methods

- DSM265 and DSM450 concentrations in both dried blood samples (DBS) and plasma for 4 patients were obtained from Swiss BioQuant.

- Using Phoenix WinNonlin (version 6.3), a noncompartmental analysis was performed. The variables t_{max} and C_{max} were directly read from the plasma concentration-time curve whereas AUC was calculated using the linear trapezoidal rule. The $t_{1/2}$ could not be estimated reliably for the metabolite DSM450.

- Tables were generated with WinNonlin whereas the mean plasma concentration-time and the 'spaghetti' curves were generated with GraphPad.

Results: Cohort 1A.

Table S1 Mean DSM265 DBS concentrations (ng/mL) and descriptive statistics following administration of a single dose of 400 mg DSM265.

Time (h)	N	Mean	SD	Min	Median	Max	CV%
0.00	4	0.00	0.00	0.00	0.00	0.00	
0.250	4	673	691	116	454	1670	102.7
0.500	4	2140	1290	354	2380	3440	60.4
1.00	4	4330	3130	675	4700	7230	72.4
2.00	4	5320	3120	1540	5330	9060	58.8
4.00	4	5680	1430	3920	5750	7310	25.2
6.00	4	5320	1370	3840	5410	6600	25.7
8.00	4	5340	1560	3960	4950	7490	29.2
14.0	4	5290	1640	3480	5480	6720	31.1
24.0	4	5160	1570	3310	5100	7140	30.3
48.0	4	4000	1130	2560	4070	5310	28.2
72.0	4	3600	911	2390	3800	4400	25.4
168	4	2210	649	1650	2050	3100	29.3
264	4	1160	246	926	1140	1430	21.2
432	4	514	348	145	463	985	67.7

Table S2 Mean DSM265 plasma concentrations (ng/mL) and descriptive statistics following administration of a single dose of 400 mg DSM265.

Time (h)	N	Mean	SD	Min	Median	Max	CV%
0.00	4	0.00	0.00	0.00	0.00	0.00	
0.250	4	1390	1220	229	1120	3080	87.8
0.500	4	3990	2490	683	4310	6650	62.4
1.00	4	8940	6730	1390	9590	15200	75.3
2.00	4	10900	6400	3360	11600	17000	58.9
4.00	4	11700	2850	7610	12700	13800	24.4
6.00	4	10800	3060	7180	11000	14000	28.4
8.00	4	10300	3650	6300	10100	14800	35.4
14.0	4	10200	3000	7410	9990	13400	29.4
24.0	4	9480	2760	6040	9800	12300	29.1
48.0	4	7200	2170	4160	7740	9160	30.1
72.0	4	6710	1480	4580	7140	8000	22.1
168	4	4190	1040	3200	3990	5580	24.7
264	4	2210	356	1850	2180	2640	16.1
432	4	973	684	304	829	1930	70.4

Table S3 Mean DSM450 DBS concentrations (ng/mL) and descriptive statistics following administration of a single dose of 400 mg DSM265.

Time (h)	N	Mean	SD	Min	Median	Max	CV%
0.00	4	0.00	0.00	0.00	0.00	0.00	
0.250	4	0.558	1.12	0.00	0.00	2.23	200.0
0.500	4	3.16	2.53	0.00	3.34	5.94	80.1
1.00	4	12.1	11.3	0.00	12.6	23.4	92.9
2.00	4	24.5	21.4	3.30	22.7	49.4	87.2
4.00	4	79.6	89.4	13.7	49.4	206	112.3
6.00	4	65.5	47.4	22.5	65.3	109	72.4
8.00	4	89.9	63.9	33.9	86.8	152	71.2
14.0	4	167	126	57.5	163	283	75.4
24.0	4	247	147	112	232	410	59.8
48.0	4	380	209	187	354	625	55.1
72.0	4	453	225	223	439	712	49.6
168	4	571	232	309	550	874	40.6
264	4	511	165	344	504	692	32.3
432	4	266	162	86.8	272	433	61.1

Table S4 Mean DSM450 plasma concentrations (ng/mL) and descriptive statistics following administration of a single dose of 400 mg DSM265.

Time (h)	N	Mean	SD	Min	Median	Max	CV%
0.00	4	0.00	0.00	0.00	0.00	0.00	
0.250	4	0.895	1.79	0.00	0.00	3.58	200.0
0.500	4	5.21	4.78	0.00	4.97	10.9	91.7
1.00	4	21.7	20.6	0.00	22.8	41.1	95.0
2.00	4	43.8	37.7	5.48	45.2	79.4	86.1
4.00	4	78.4	56.4	23.7	79.9	130	72.0
6.00	4	118	82.3	46.2	118	190	69.7
8.00	4	162	119	54.9	160	272	73.8
14.0	4	302	226	96.5	287	538	74.9
24.0	4	458	288	198	463	707	62.9
48.0	4	690	416	290	685	1100	60.3
72.0	4	830	440	405	813	1290	53.0
168	4	1020	369	589	996	1490	36.3
264	4	904	245	663	891	1170	27.1
432	4	429	254	156	434	692	59.3

Figure S1 Mean DSM265 and DSM450 concentration-time profiles in both DBS and plasma following administration of a single dose of 400 mg DSM265.

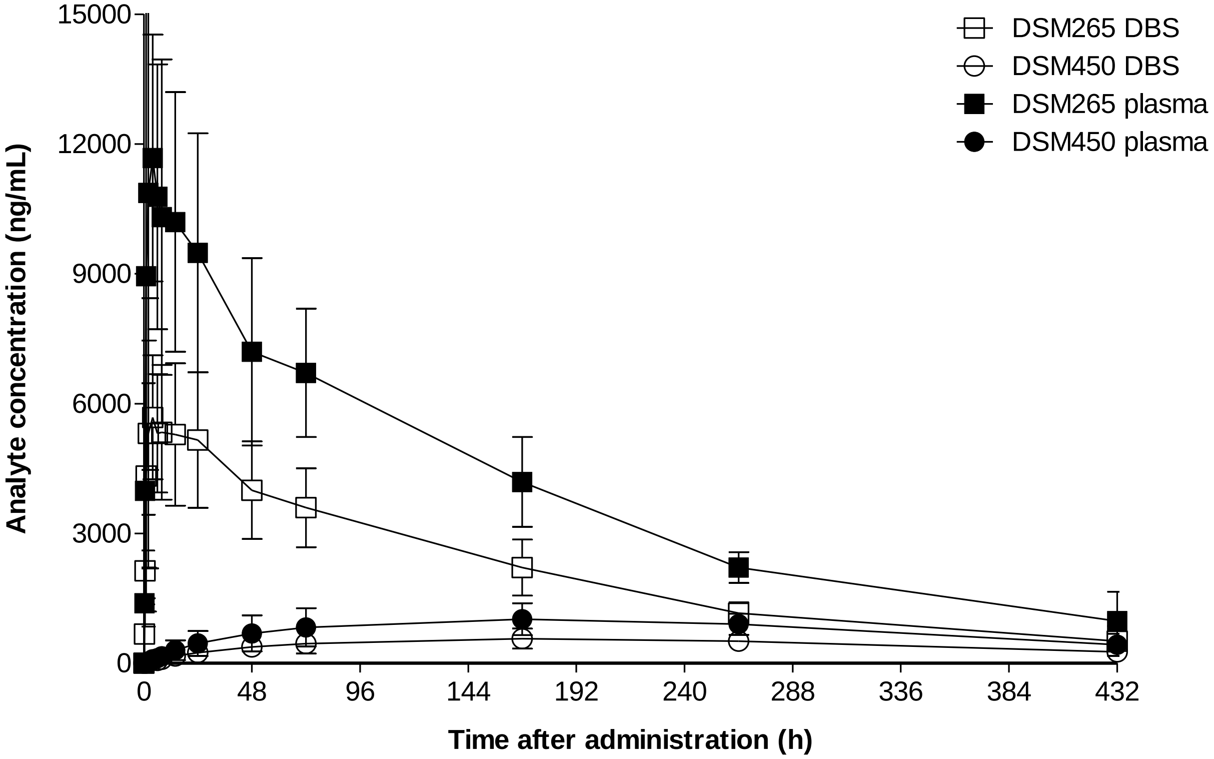

Figure S2 Individual (spaghetti) concentration-time profiles in both DBS and plasma of DSM265 and DSM450 following administration of a single dose of 400 mg DSM265

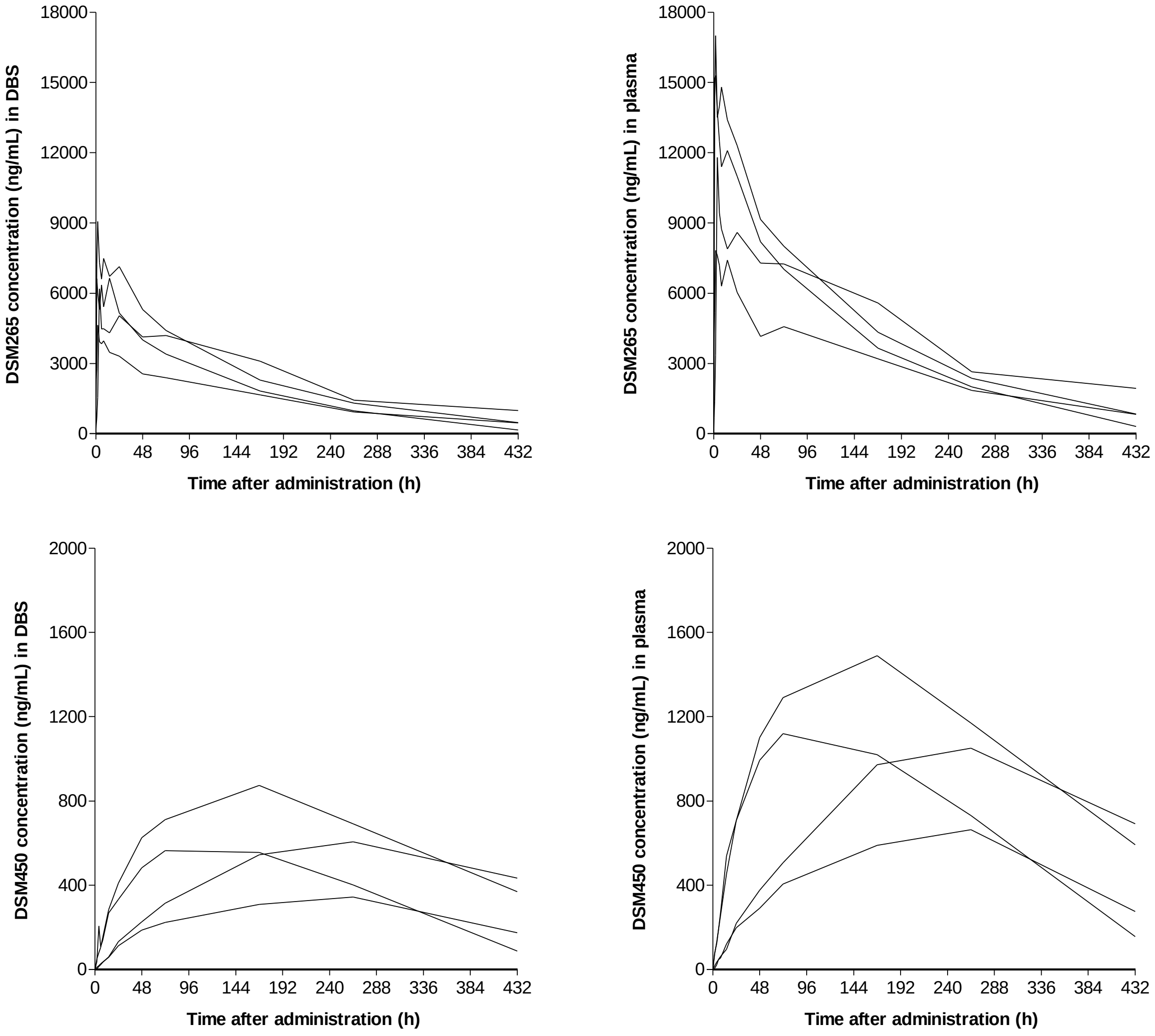

Table S5 Individual and mean DSM265 DBS pharmacokinetic variables following administration of single doses of 400 mg DSM265.

patient	t_{max} (h)	C_{max} (ng/mL)	$AUC_{0\text{-}t}$ (ng.h/mL)	$AUC_{0\text{-}\infty}$ (ng.h/mL)	$AUC_{0\text{-}168h}$ (ng.h/mL)	$t_{1/2}$ (h)
SBQ-0113	4.00	6190	1080000	1320000	662000	170
SBQ-0118	14.0	6640	816000	834000	588000	82.0
SBQ-0123	2.00	4630	650000	750000	410000	151
SBQ-0144	2.00	9060	1070000	1150000	755000	110
N	4	4	4	4	4	4
Mean	5.50	6630	906000	1010000	603000	128
SD	5.74	1830	211000	268000	146000	39.6
Min	2.00	4630	650000	750000	410000	82.0
Median	3.00	6420	946000	991000	625000	131
Max	14.0	9060	1080000	1320000	755000	170
Geometric Mean	3.87	6440	886000	987000	589000	123
CV% Geometric Mean	114.9	28.1	24.9	27.1	26.7	33.7

Table S6 Individual and mean DSM265 plasma pharmacokinetic variables following administration of single doses of 400 mg DSM265.

patient	t_{max} (h)	C_{max} (ng/mL)	$AUC_{0\text{-}t}$ (ng.h/mL)	$AUC_{0\text{-}\infty}$ (ng.h/mL)	$AUC_{0\text{-}168h}$ (ng.h/mL)	$t_{1/2}$ (h)
SBQ-0113	4.00	11800	1950000	2460000	1170000	183
SBQ-0118	2.00	15300	1680000	1710000	1210000	82.5
SBQ-0123	2.00	7820	1230000	1410000	762000	155
SBQ-0144	2.00	17000	1970000	2100000	1380000	111
N	4	4	4	4	4	4
Mean	2.50	13000	1710000	1920000	1130000	133
SD	1.00	4060	344000	455000	261000	44.9
Min	2.00	7820	1230000	1410000	762000	82.5
Median	2.00	13600	1810000	1910000	1190000	133
Max	4.00	17000	1970000	2460000	1380000	183
Geometric Mean	2.38	12400	1680000	1880000	1100000	127
CV% Geometric Mean	35.7	35.6	22.2	24.4	26.1	36.7

Table S7 Individual and mean DSM450 DBS pharmacokinetic variables following administration of single doses of 400 mg DSM265.

patient	t_{max} (h)	C_{max} (ng/mL)	AUC_{0-t} (ng.h/mL)	AUC_{0-168h} (ng.h/mL)
SBQ-0113	264	606	196000	53300
SBQ-0118	72.0	564	168000	81100
SBQ-0123	264	344	110000	35300
SBQ-0144	168	874	274000	110000
N	4	4	4	4
Mean	192	597	187000	69900
SD	91.9	217	68200	32700
Min	72.0	344	110000	35300
Median	216	585	182000	67200
Max	264	874	274000	110000
Geometric Mean	170	566	178000	64000
CV% Geometric Mean	67.5	39.8	39.2	52.7

Table S8 Individual and mean DSM450 plasma pharmacokinetic variables following administration of single doses of 400 mg DSM265.

patient	t_{max} (h)	C_{max} (ng/mL)	AUC_{0-t} (ng.h/mL)	AUC_{0-168h} (ng.h/mL)
SBQ-0113	264	1050	334000	90900
SBQ-0118	72.0	1120	316000	157000
SBQ-0123	264	663	203000	64300
SBQ-0144	168	1490	469000	194000
N	4	4	4	4
Mean	192	1080	331000	127000
SD	91.9	339	109000	59400
Min	72.0	663	203000	64300
Median	216	1090	325000	124000
Max	264	1490	469000	194000
Geometric Mean	170	1040	317000	116000
CV% Geometric Mean	67.5	34.5	35.4	53.8

Figure S3 Correlation between DBS and plasma concentrations of DSM265 and DSM450

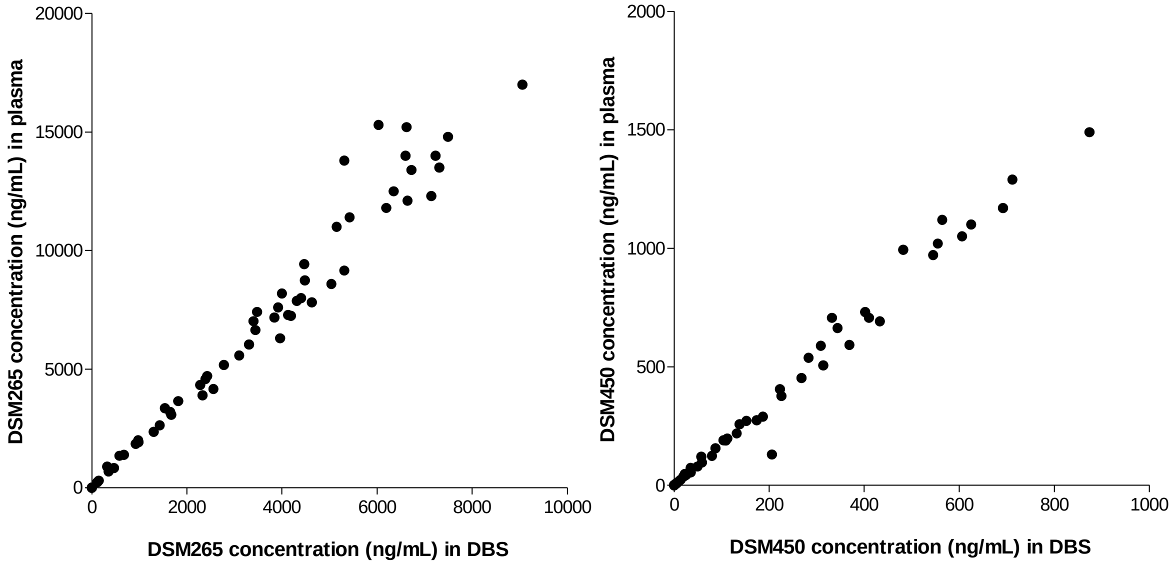

Results: Cohort 2

Table S9. Cohort 2: Mean DSM265 DBS concentrations (ng/mL) and descriptive statistics following administration of a single dose of 400 mg DSM265

Time (h)	N	Mean	SD	Min	Median	Max	CV%
0.00	6	0.00	0.00	0.00	0.00	0.00	
0.250	6	1140	650	319	1010	2210	57.2
0.500	6	3260	2470	1350	2600	7970	75.8
1.00	6	4060	2210	1550	3760	6990	54.3
2.00	6	4340	1860	1190	4630	6190	42.8
4.00	6	4770	1700	2270	5050	7310	35.6
6.00	6	5090	1610	3120	5270	6960	31.6
8.00	6	5560	1540	3750	5640	7070	27.7
14.0	6	6020	1400	4490	5710	8410	23.2
24.0	6	5040	867	3820	5350	5940	17.2
48.0	6	4110	612	3290	4030	4880	14.9
72.0	6	3380	571	2770	3260	4320	16.9
168	6	1900	650	1080	1960	2740	34.3
264	6	1230	670	337	1320	2280	54.6
480	6	343	237	19.4	383	671	69.1

Table S10 Cohort 2: Mean DSM265 plasma concentrations (ng/mL) and descriptive statistics following administration of a single dose of 400 mg DSM265.

Time (h)	N	Mean	SD	Min	Median	Max	CV%
0.00	6	0.00	0.00	0.00	0.00	0.00	
0.250	6	2020	1130	536	1740	3960	56.0
0.500	6	5450	4400	2440	4060	14100	80.8
1.00	6	6620	3460	2190	6630	11500	52.3
2.00	6	7080	3050	1940	7710	9850	43.1
4.00	6	7770	3090	3490	7920	12400	39.8
6.00	6	8160	2630	4950	8590	10700	32.3
8.00	6	8450	2250	5760	8640	10600	26.6
14.0	6	9710	1780	7360	9540	12800	18.3
24.0	6	8170	904	6980	8170	9270	11.1
48.0	6	6510	1310	4940	6210	8210	20.1
72.0	6	5810	1050	4840	5480	7440	18.1
168	6	3400	1150	1760	3660	4650	34.0
264	6	2020	1070	540	2140	3640	53.2
480	6	612	404	36.8	752	1090	66.1

Table S11 Cohort 2: Mean DSM450 DBS concentrations (ng/mL) and descriptive statistics following administration of a single dose of 400 mg DSM265

Time (h)	N	Mean	SD	Min	Median	Max	CV%
0.00	6	0.00	0.00	0.00	0.00	0.00	
0.250	6	2.07	2.31	0.00	1.79	4.94	111.7
0.500	6	9.03	9.77	0.00	6.78	28.2	108.2
1.00	6	21.9	17.6	4.04	18.6	49.3	80.5
2.00	6	43.4	36.7	5.27	31.7	101	84.5
4.00	6	72.8	49.0	15.2	64.6	150	67.3
6.00	6	101	63.4	21.3	92.7	182	62.8
8.00	6	132	76.6	33.3	132	221	57.9
14.0	6	219	127	75.8	198	410	57.9
24.0	6	316	132	153	296	502	41.7
48.0	6	541	212	293	549	822	39.2
72.0	6	629	311	332	551	1160	49.4
168	6	701	342	442	589	1350	48.8
264	6	611	375	317	514	1360	61.5
480	6	265	189	39.1	236	574	71.3

Table S12 Cohort 2: Mean DSM450 plasma concentrations (ng/mL) and descriptive statistics following administration of a single dose of 400 mg DSM265

Time (h)	N	Mean	SD	Min	Median	Max	CV%
0.00	6	0.00	0.00	0.00	0.00	0.00	
0.250	6	3.03	3.58	0.00	1.82	7.65	118.2
0.500	6	14.8	16.4	2.41	9.80	47.6	110.4
1.00	6	33.8	29.2	4.80	24.5	78.8	86.2
2.00	6	66.0	52.6	8.08	51.3	143	79.6
4.00	6	112	69.3	21.5	108	207	61.8
6.00	6	153	92.1	35.4	150	258	60.1
8.00	6	189	106	50.3	188	312	56.0
14.0	6	334	176	115	343	548	52.9
24.0	6	522	261	236	494	940	50.0
48.0	6	825	346	434	814	1300	41.9
72.0	6	1030	408	555	985	1650	39.7
168	6	1200	515	758	1020	2190	42.8
264	6	929	540	426	787	1990	58.2
480	6	435	308	64.0	403	954	70.8

Figure S4 MMV_DSM265_14_01 Cohort 2: Mean DSM265 and DSM450 concentration-time profiles in both DBS and plasma following administration of a single dose of 400 mg DSM265

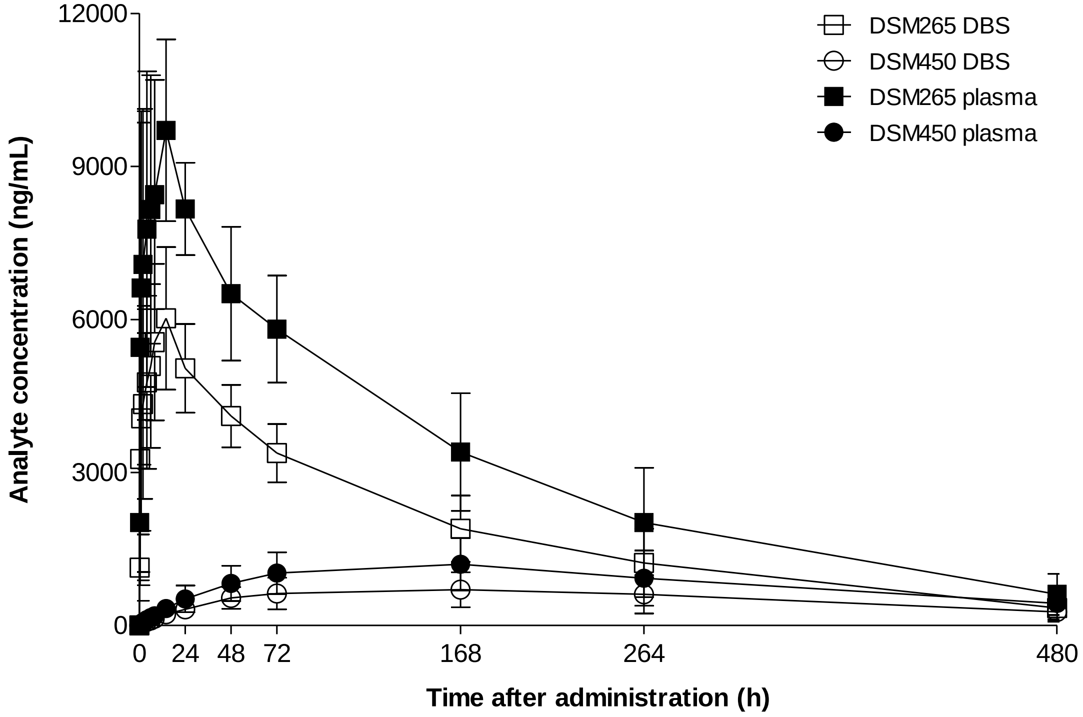

Figure S5 Cohort 2: Individual (spaghetti) concentration-time profiles in both DBS and plasma of DSM265 and DSM450 following administration of a single dose of 400 mg DSM265

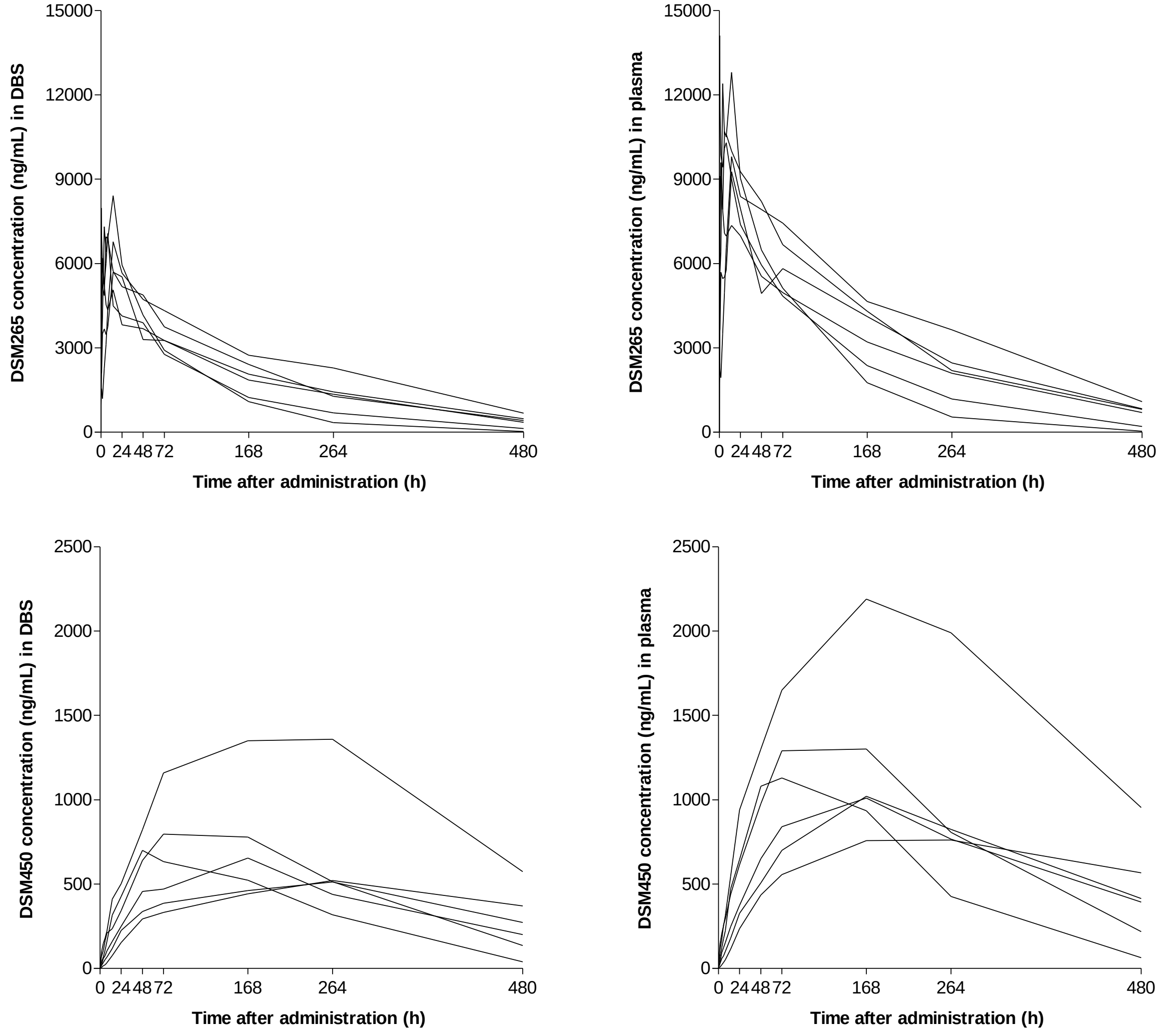

Table S13 Cohort 2: Individual and mean DSM265 DBS pharmacokinetic variables following administration of single doses of 400 mg DSM265

Subject	t_{max} (h)	C_{max} (ng/mL)	AUC_{0-t} (ng.h/mL)	$AUC_{0-\infty}$ (ng.h/mL)	AUC_{0-168h} (ng.h/mL)	$t_{1/2}$ (h)
SBQ-0213	4.00	7310	1020000	1090000	657000	127
SBQ-0221	0.500	7970	674000	691000	493000	93.1
SBQ-0244	14.0	8410	667000	668000	560000	55.9
SBQ-0269	2.00	6190	867000	932000	529000	128
SBQ-0273	14.0	5690	923000	1020000	550000	147
SBQ-0285	14.0	6770	1250000	1400000	686000	154
N	6	6	6	6	6	6
Mean	8.08	7060	899000	967000	579000	118
SD	6.58	1040	219000	272000	75700	36.9
Min	0.500	5690	667000	668000	493000	55.9
Median	9.00	7040	895000	978000	555000	128
Max	14.0	8410	1250000	1400000	686000	154
Geometric Mean	4.71	6990	877000	936000	575000	111
CV% Geometric Mean	234.3	15.0	24.6	28.7	12.9	39.6

Table S14 Cohort 2: Individual and mean DSM265 plasma pharmacokinetic variables following administration of single doses of 400 mg DSM265

Subject	t_{max} (h)	C_{max} (ng/mL)	AUC_{0-t} (ng.h/mL)	$AUC_{0-\infty}$ (ng.h/mL)	AUC_{0-168h} (ng.h/mL)	$t_{1/2}$ (h)
SBQ-0213	4.00	12400	1780000	1940000	1150000	132
SBQ-0221	0.500	14100	1170000	1200000	854000	89.8
SBQ-0244	14.0	12800	1080000	1080000	905000	57.0
SBQ-0269	2.00	9590	1400000	1540000	842000	143
SBQ-0273	14.0	9260	1600000	1780000	932000	143
SBQ-0285	14.0	9810	2040000	2270000	1130000	149
N	6	6	6	6	6	6
Mean	8.08	11300	1510000	1630000	968000	119
SD	6.58	2030	367000	452000	135000	37.2
Min	0.500	9260	1080000	1080000	842000	57.0
Median	9.00	11100	1500000	1660000	919000	138
Max	14.0	14100	2040000	2270000	1150000	149
Geometric Mean	4.71	11200	1480000	1580000	961000	113
CV% Geometric Mean	234.3	18.0	24.9	29.1	13.7	39.8

Table S15 Individual and mean DSM450 DBS pharmacokinetic variables following administration of single doses of 400 mg DSM265.

Subject	t_{max} (h)	C_{max} (ng/mL)	AUC_{0-t} (ng.h/mL)	AUC_{0-168h} (ng.h/mL)
SBQ-0213	168	655	198000	76800
SBQ-0221	72.0	795	242000	110000
SBQ-0244	48.0	700	169000	90600
SBQ-0269	264	1360	507000	168000
SBQ-0273	264	513	190000	58600
SBQ-0285	264	521	194000	51600
N	6	6	6	6
Mean	180	757	250000	92500
SD	100	314	128000	42500
Min	48.0	513	169000	51600
Median	216	678	196000	83700
Max	264	1360	507000	168000
Geometric Mean	148	714	231000	85400
CV% Geometric Mean	86.8	37.1	41.8	45.2

Table S16 Cohort 2: Individual and mean DSM450 plasma pharmacokinetic variables following administration of single doses of 400 mg DSM265

Subject	t_{max} (h)	C_{max} (ng/mL)	AUC_{0-t} (ng.h/mL)	AUC_{0-168h} (ng.h/mL)
SBQ-0213	168	1010	334000	124000
SBQ-0221	168	1300	391000	179000
SBQ-0244	72.0	1130	273000	155000
SBQ-0269	168	2190	777000	258000
SBQ-0273	168	1020	333000	111000
SBQ-0285	264	760	302000	85400
N	6	6	6	6
Mean	168	1240	402000	152000
SD	60.7	500	188000	61600
Min	72.0	760	273000	85400
Median	168	1080	334000	139000
Max	264	2190	777000	258000
Geometric Mean	157	1170	375000	143000
CV% Geometric Mean	44.3	36.7	39.0	40.5

Figure S6 MMV_DSM265_14_01 Cohort 2: Correlation between DBS and plasma concentrations of DSM265 and DSM450

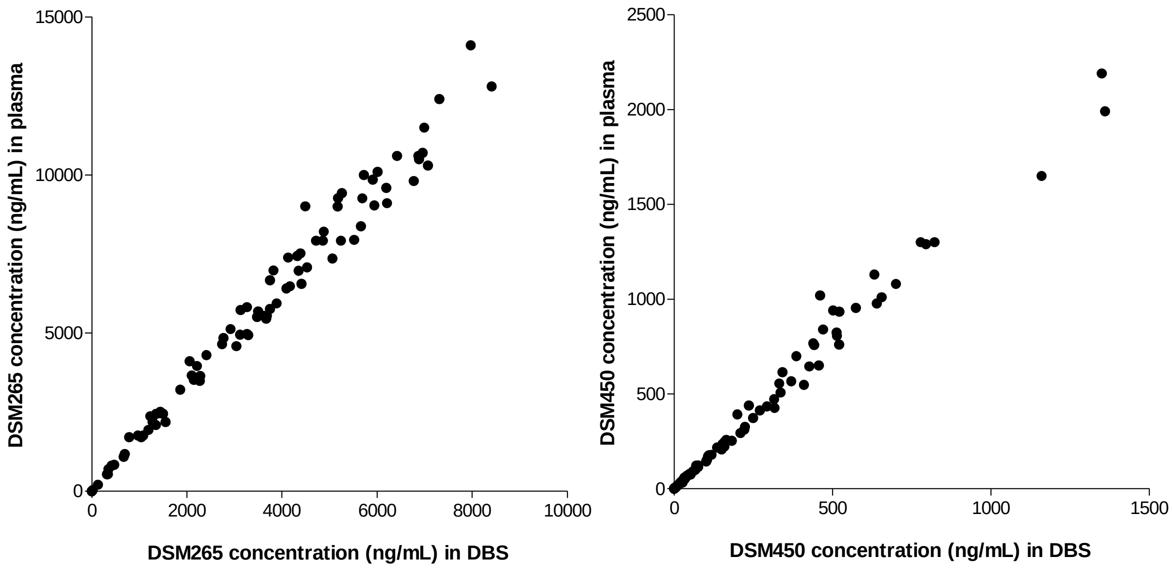

Microscopically detectable parasitaemia in Cohort 2

Figure S7. Kaplan-Meyer curves of Cohort 2. Blue curve: placebo, red curve: DSM265. Endpoint microscopically detectable parasitaemia (log-rank test: p = 0·036).

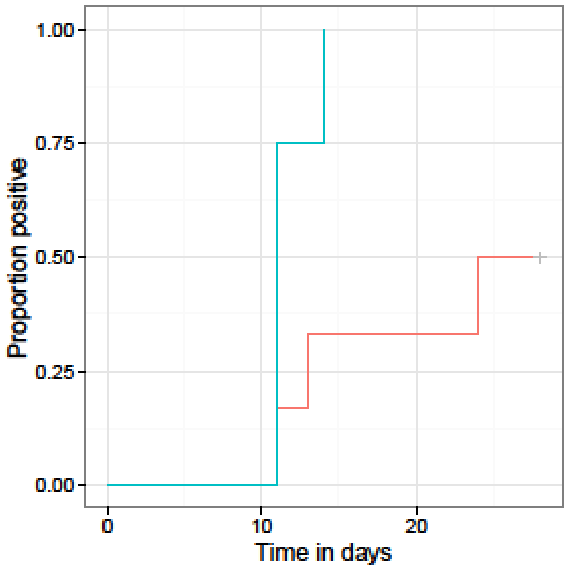

PK/PD relationship of Cohort 1A and 2.

Table S17. PK/PD relationship of Cohort 1A and 2.

Cohort 1A							
Participant	1A-003	1A-005	1A-008	1A-001	1A-002	1A-006	1A-007
DSM265 blood concentration at moment of PfSPZ inoculum[ng/mL]	3310	5040	5150	7140	0	0	ND*
TBS positive [day post DVI]	NA	NA	NA	NA	11	14	NA

Cohort 2								
Participant	2-201	2-202	2-203	2-204	2-205	2-206	2-207	2-208
DSM265 blood concentration at moment of PfSPZ inoculum [ng/mL]	2740	2410	0	0	1860	1230	2060	1080
TBS positive [day post DVI]	NA	24	11	11	NA	13	NA	11

TBS: thick blood smear; DVI: direct venous inoculation, NA: not determined

Treatment emergent adverse events

From 1st treatment administration up to End of Study Visit (Day 60)

System Organ Class Preferred Term	Grade	DSM265 Cohort 1A (n=5)	Malarone Cohort 1B (n=6)	DSM265 Cohort 2 (n=6)	DSM265 Cohorts 1A and 2 (n=11)	Placebo Cohorts 1A and 2 (n=4)
**Subjects with any unrelated TEAE**	Any grade	19 / 4 (80.0%)	16 / 5 (83.3%)	77 / 6 (100.0%)	96 / 10 (90.9%)	50 / 4 (100.0%)
	Grade 1	16 / 4 (80.0%)	15 / 5 (83.3%)	57 / 6 (100.0%)	73 / 10 (90.9%)	41 / 4 (100.0%)
	Grade 2		1 / 1 (16.7%)	13 / 4 (66.7%)	13 / 4 (36.4%)	8 / 3 (75.0%)
	Grade 3	1 / 1 (20.0%)		5 / 3 (50.0%)	6 / 4 (36.4%)	1 / 1 (25.0%)
	Grade 4	2 / 1 (20.0%)		2 / 1 (16.7%)	4 / 2 (18.2%)	
**Blood and lymphatic system disorders**				8 / 3 (50.0%)	8 / 3 (27.3%)	3 / 2 (50.0%)
Haemolysis	Any grade			1 / 1 (16.7%)	1 / 1 (9.1%)	1 / 1 (25.0%)
	Grade 1			1 / 1 (16.7%)	1 / 1 (9.1%)	1 / 1 (25.0%)
Leukopenia	Any grade			1 / 1 (16.7%)	1 / 1 (9.1%)	1 / 1 (25.0%)
	Grade 1			1 / 1 (16.7%)	1 / 1 (9.1%)	
	Grade 2					1 / 1 (25.0%)
Lymphadenopathy	Any grade			1 / 1 (16.7%)	1 / 1 (9.1%)	
	Grade 1			1 / 1 (16.7%)	1 / 1 (9.1%)	
Lymphopenia	Any grade			3 / 2 (33.3%)	3 / 2 (18.2%)	
	Grade 1			1 / 1 (16.7%)	1 / 1 (9.1%)	
	Grade 2			1 / 1 (16.7%)	1 / 1 (9.1%)	
	Grade 4			1 / 1 (16.7%)	1 / 1 (9.1%)	
Monocytosis	Any grade					1 / 1 (25.0%)
	Grade 1					1 / 1 (25.0%)
Neutropenia	Any grade			1 / 1 (16.7%)	1 / 1 (9.1%)	

System Organ Class Preferred Term	Grade	DSM265 Cohort 1A (n=5)	Malarone Cohort 1B (n=6)	DSM265 Cohort 2 (n=6)	DSM265 Cohorts 1A and 2 (n=11)	Placebo Cohorts 1A and 2 (n=4)
Neutropenia	Grade 1			1 / 1 (16.7%)	1 / 1 (9.1%)	
Thrombocytopenia	Any grade			1 / 1 (16.7%)	1 / 1 (9.1%)	
	Grade 1			1 / 1 (16.7%)	1 / 1 (9.1%)	
**Cardiac disorders**		2 / 1 (20.0%)		5 / 4 (66.7%)	7 / 5 (45.5%)	3 / 2 (50.0%)
Palpitations	Any grade	1 / 1 (20.0%)			1 / 1 (9.1%)	1 / 1 (25.0%)
	Grade 1	1 / 1 (20.0%)			1 / 1 (9.1%)	1 / 1 (25.0%)
Sinus tachycardia	Any grade	1 / 1 (20.0%)			1 / 1 (9.1%)	
	Grade 1	1 / 1 (20.0%)			1 / 1 (9.1%)	
Tachycardia	Any grade			5 / 4 (66.7%)	5 / 4 (36.4%)	2 / 2 (50.0%)
	Grade 1			5 / 4 (66.7%)	5 / 4 (36.4%)	2 / 2 (50.0%)
**Ear and labyrinth disorders**						1 / 1 (25.0%)
Ear pain	Any grade					1 / 1 (25.0%)
	Grade 1					1 / 1 (25.0%)

System Organ Class Preferred Term	Grade	DSM265 Cohort 1A (n=5)	Malarone Cohort 1B (n=6)	DSM265 Cohort 2 (n=6)	DSM265 Cohorts 1A and 2 (n=11)	Placebo Cohorts 1A and 2 (n=4)
Eye disorders				1 / 1 (16.7%)	1 / 1 (9.1%)	
Eye pain	Any grade			1 / 1 (16.7%)	1 / 1 (9.1%)	
	Grade 1			1 / 1 (16.7%)	1 / 1 (9.1%)	
**Gastrointestinal disorders**			3 / 1 (16.7%)	4 / 3 (50.0%)	4 / 3 (27.3%)	1 / 1 (25.0%)
Abdominal pain	Any grade			2 / 1 (16.7%)	2 / 1 (9.1%)	
	Grade 1			2 / 1 (16.7%)	2 / 1 (9.1%)	
Aphthous ulcer	Any grade		1 / 1 (16.7%)			
Aphthous ulcer	Grade 1		1 / 1 (16.7%)			
Constipation	Any grade		2 / 1 (16.7%)			
	Grade 1		2 / 1 (16.7%)			
Nausea	Any grade			1 / 1 (16.7%)	1 / 1 (9.1%)	1 / 1 (25.0%)
	Grade 1			1 / 1 (16.7%)	1 / 1 (9.1%)	1 / 1 (25.0%)
Vomiting	Any grade			1 / 1 (16.7%)	1 / 1 (9.1%)	
	Grade 1			1 / 1 (16.7%)	1 / 1 (9.1%)	
**General disorders and administration site conditions**		1 / 1 (20.0%)	2 / 2 (33.3%)	21 / 6 (100.0%)	22 / 7 (63.6%)	18 / 4 (100.0%)

System Organ Class Preferred Term	Grade	DSM265 Cohort 1A (n=5)	Malarone Cohort 1B (n=6)	DSM265 Cohort 2 (n=6)	DSM265 Cohorts 1A and 2 (n=11)	Placebo Cohorts 1A and 2 (n=4)
Chest discomfort	Any grade					1 / 1 (25.0%)
	Grade 1					1 / 1 (25.0%)
Chills	Any grade		1 / 1 (16.7%)	3 / 3 (50.0%)	3 / 3 (27.3%)	2 / 2 (50.0%)
	Grade 1		1 / 1 (16.7%)	3 / 3 (50.0%)	3 / 3 (27.3%)	2 / 2 (50.0%)
Fatigue	Any grade	1 / 1 (20.0%)	1 / 1 (16.7%)	10 / 6 (100.0%)	11 / 7 (63.6%)	9 / 4 (100.0%)
	Grade 1	1 / 1 (20.0%)	1 / 1 (16.7%)	8 / 6 (100.0%)	9 / 7 (63.6%)	7 / 4 (100.0%)
	Grade 2			2 / 2 (33.3%)	2 / 2 (18.2%)	1 / 1 (25.0%)
	Grade 3					1 / 1 (25.0%)
Pyrexia	Any grade			8 / 3 (50.0%)	8 / 3 (27.3%)	6 / 2 (50.0%)
	Grade 1			1 / 1 (16.7%)	1 / 1 (9.1%)	3 / 2 (50.0%)
	Grade 2			3 / 3 (50.0%)	3 / 3 (27.3%)	3 / 2 (50.0%)
	Grade 3			4 / 2 (33.3%)	4 / 2 (18.2%)	
**Hepatobiliary disorders**				1 / 1 (16.7%)	1 / 1 (9.1%)	
Hyperbilirubinemia	Any grade			1 / 1 (16.7%)	1 / 1 (9.1%)	
	Grade 1			1 / 1 (16.7%)	1 / 1 (9.1%)	
**Infections and infestations**		2 / 2 (40.0%)	4 / 3 (50.0%)	3 / 3 (50.0%)	5 / 5 (45.5%)	3 / 1 (25.0%)
Nasopharyngitis	Any grade			1 / 1 (16.7%)	1 / 1 (9.1%)	1 / 1 (25.0%)
	Grade 1			1 / 1 (16.7%)	1 / 1 (9.1%)	1 / 1 (25.0%)
Rhinitis	Any grade	1 / 1 (20.0%)			1 / 1 (9.1%)	
	Grade 1	1 / 1 (20.0%)			1 / 1 (9.1%)	

System Organ Class Preferred Term	Grade	DSM265 Cohort 1A (n=5)	Malarone Cohort 1B (n=6)	DSM265 Cohort 2 (n=6)	DSM265 Cohorts 1A and 2 (n=11)	Placebo Cohorts 1A and 2 (n=4)
Sinusitis	Any grade			2 / 2 (33.3%)	2 / 2 (18.2%)	1 / 1 (25.0%)
	Grade 1			2 / 2 (33.3%)	2 / 2 (18.2%)	1 / 1 (25.0%)
Tonsillitis	Any grade					1 / 1 (25.0%)
	Grade 1					1 / 1 (25.0%)
Upper respiratory tract infection	Any grade		2 / 1 (16.7%)			
	Grade 1		2 / 1 (16.7%)			
Viral upper respiratory tract infection	Any grade	1 / 1 (20.0%)	2 / 2 (33.3%)		1 / 1 (9.1%)	
	Grade 1	1 / 1 (20.0%)	2 / 2 (33.3%)		1 / 1 (9.1%)	
**Investigations**		1 / 1 (20.0%)		5 / 3 (50.0%)	6 / 4 (36.4%)	
Alanine aminotransferase increased	Any grade			2 / 2 (33.3%)	2 / 2 (18.2%)	
	Grade 1			1 / 1 (16.7%)	1 / 1 (9.1%)	
	Grade 2			1 / 1 (16.7%)	1 / 1 (9.1%)	
Aspartate aminotransferase increased	Any grade			2 / 1 (16.7%)	2 / 1 (9.1%)	
	Grade 1			1 / 1 (16.7%)	1 / 1 (9.1%)	
	Grade 2			1 / 1 (16.7%)	1 / 1 (9.1%)	
Blood pressure systolic increased	Any grade			1 / 1 (16.7%)	1 / 1 (9.1%)	
	Grade 2			1 / 1 (16.7%)	1 / 1 (9.1%)	

System Organ Class Preferred Term	Grade	DSM265 Cohort 1A (n=5)	Malarone Cohort 1B (n=6)	DSM265 Cohort 2 (n=6)	DSM265 Cohorts 1A and 2 (n=11)	Placebo Cohorts 1A and 2 (n=4)
Cardiac murmur	Any grade	1 / 1 (20.0%)			1 / 1 (9.1%)	
	Grade 1	1 / 1 (20.0%)			1 / 1 (9.1%)	
**Metabolism and nutrition disorders**			1 / 1 (16.7%)	2 / 2 (33.3%)	2 / 2 (18.2%)	
Hyperkalaemia	Any grade			2 / 2 (33.3%)	2 / 2 (18.2%)	
	Grade 3			1 / 1 (16.7%)	1 / 1 (9.1%)	
	Grade 4			1 / 1 (16.7%)	1 / 1 (9.1%)	
Hypoglycaemia	Any grade		1 / 1 (16.7%)			
	Grade 2		1 / 1 (16.7%)			
**Musculoskeletal and connective tissue disorders**		1 / 1 (20.0%)		5 / 4 (66.7%)	6 / 5 (45.5%)	4 / 2 (50.0%)
Flank pain	Any grade					1 / 1 (25.0%)
	Grade 1					1 / 1 (25.0%)
Myalgia	Any grade	1 / 1 (20.0%)		5 / 4 (66.7%)	6 / 5 (45.5%)	3 / 2 (50.0%)
	Grade 1	1 / 1 (20.0%)		4 / 4 (66.7%)	5 / 5 (45.5%)	3 / 2 (50.0%)
	Grade 2			1 / 1 (16.7%)	1 / 1 (9.1%)	
**Nervous system disorders**		4 / 2 (40.0%)		11 / 5 (83.3%)	15 / 7 (63.6%)	12 / 4 (100.0%)
Dizziness	Any grade	1 / 1 (20.0%)			1 / 1 (9.1%)	2 / 2 (50.0%)
	Grade 1	1 / 1 (20.0%)			1 / 1 (9.1%)	2 / 2 (50.0%)
Headache	Any grade	2 / 1 (20.0%)		11 / 5 (83.3%)	13 / 6 (54.5%)	10 / 4 (100.0%)
	Grade 1	2 / 1 (20.0%)		8 / 5 (83.3%)	10 / 6 (54.5%)	7 / 3 (75.0%)

System Organ Class Preferred Term	Grade	DSM265 Cohort 1A (n=5)	Malarone Cohort 1B (n=6)	DSM265 Cohort 2 (n=6)	DSM265 Cohorts 1A and 2 (n=11)	Placebo Cohorts 1A and 2 (n=4)
	Grade 2			3 / 3 (50.0%)	3 / 3 (27.3%)	3 / 3 (75.0%)
Loss of consciousness	Any grade	1 / 1 (20.0%)			1 / 1 (9.1%)	
	Grade 4	1 / 1 (20.0%)			1 / 1 (9.1%)	
**Psychiatric disorders**		2 / 1 (20.0%)		1 / 1 (16.7%)	3 / 2 (18.2%)	1 / 1 (25.0%)
Abnormal dreams	Any grade			1 / 1 (16.7%)	1 / 1 (9.1%)	
	Grade 1			1 / 1 (16.7%)	1 / 1 (9.1%)	
Insomnia	Any grade					1 / 1 (25.0%)
	Grade 1					1 / 1 (25.0%)
Sleep disorder	Any grade	2 / 1 (20.0%)			2 / 1 (9.1%)	
	Grade 1	2 / 1 (20.0%)			2 / 1 (9.1%)	
**Respiratory, thoracic and mediastinal disorders**		4 / 2 (40.0%)	4 / 2 (33.3%)	6 / 2 (33.3%)	10 / 4 (36.4%)	2 / 2 (50.0%)
Cough	Any grade	1 / 1 (20.0%)		2 / 2 (33.3%)	3 / 3 (27.3%)	1 / 1 (25.0%)
	Grade 1	1 / 1 (20.0%)		2 / 2 (33.3%)	3 / 3 (27.3%)	1 / 1 (25.0%)
Dysphonia	Any grade		1 / 1 (16.7%)	1 / 1 (16.7%)	1 / 1 (9.1%)	
	Grade 1		1 / 1 (16.7%)	1 / 1 (16.7%)	1 / 1 (9.1%)	
Dyspnoea	Any grade	1 / 1 (20.0%)		1 / 1 (16.7%)	2 / 2 (18.2%)	1 / 1 (25.0%)
	Grade 1	1 / 1 (20.0%)		1 / 1 (16.7%)	2 / 2 (18.2%)	1 / 1 (25.0%)
Nasal congestion	Any grade		1 / 1 (16.7%)	1 / 1 (16.7%)	1 / 1 (9.1%)	
	Grade 1		1 / 1 (16.7%)	1 / 1 (16.7%)	1 / 1 (9.1%)	
Oropharyngeal pain	Any grade	1 / 1 (20.0%)	2 / 2 (33.3%)	1 / 1 (16.7%)	2 / 2 (18.2%)	

System Organ Class Preferred Term	Grade	DSM265 Cohort 1A (n=5)	Malarone Cohort 1B (n=6)	DSM265 Cohort 2 (n=6)	DSM265 Cohorts 1A and 2 (n=11)	Placebo Cohorts 1A and 2 (n=4)
	Grade 1	1 / 1 (20.0%)	2 / 2 (33.3%)	1 / 1 (16.7%)	2 / 2 (18.2%)	
Pulmonary embolism	Any grade	1 / 1 (20.0%)			1 / 1 (9.1%)	
	Grade 4	1 / 1 (20.0%)			1 / 1 (9.1%)	
**Skin and subcutaneous tissue disorders**		1 / 1 (20.0%)	2 / 2 (33.3%)	3 / 3 (50.0%)	4 / 4 (36.4%)	2 / 1 (25.0%)
Acne	Any grade	1 / 1 (20.0%)			1 / 1 (9.1%)	
	Grade 1	1 / 1 (20.0%)			1 / 1 (9.1%)	
Hyperhidrosis	Any grade		1 / 1 (16.7%)	3 / 3 (50.0%)	3 / 3 (27.3%)	1 / 1 (25.0%)
	Grade 1		1 / 1 (16.7%)	3 / 3 (50.0%)	3 / 3 (27.3%)	1 / 1 (25.0%)
Night sweats	Any grade					1 / 1 (25.0%)
	Grade 1					1 / 1 (25.0%)
Urticaria	Any grade		1 / 1 (16.7%)			
	Grade 1		1 / 1 (16.7%)			
**Vascular disorders**		1 / 1 (20.0%)		1 / 1 (16.7%)	2 / 2 (18.2%)	
Deep vein thrombosis	Any grade	1 / 1 (20.0%)			1 / 1 (9.1%)	
	Grade 3	1 / 1 (20.0%)			1 / 1 (9.1%)	
Hypertension	Any grade			1 / 1 (16.7%)	1 / 1 (9.1%)	
Hypertension	Grade 1			1 / 1 (16.7%)	1 / 1 (9.1%)	

From treatment administration to initiation of the PfSPZ challenge

System Organ Class<br>Preferred Term	Grade	DSM265 Cohort 1A (n=5)	Malarone Cohort 1B (n=6)	DSM265 Cohort 2 (n=6)	DSM265 Cohorts 1A and 2 (n=11)	Placebo Cohorts 1A and 2 (n=4)
**Subjects with any unrelated TEAE**	Any grade	1 / 1 (20.0%)		11 / 5 (83.3%)	12 / 6 (54.5%)	4 / 3 (75.0%)
	Grade 1	1 / 1 (20.0%)		10 / 4 (66.7%)	11 / 5 (45.5%)	4 / 3 (75.0%)
	Grade 2			1 / 1 (16.7%)	1 / 1 (9.1%)	
**Blood and lymphatic system disorders**						2 / 1 (25.0%)
	Any grade			1 / 1 (16.7%)	1 / 1 (9.1%)	
**Cardiac disorders**				1 / 1 (16.7%)	1 / 1 (9.1%)	
Tachycardia	Any grade			1 / 1 (16.7%)	1 / 1 (9.1%)	
	Grade 1			1 / 1 (16.7%)	1 / 1 (9.1%)	
**General disorders and administration site conditions**				5 / 4 (66.7%)	5 / 4 (36.4%)	1 / 1 (25.0%)
Chills	Any grade			1 / 1 (16.7%)	1 / 1 (9.1%)	
	Grade 1			1 / 1 (16.7%)	1 / 1 (9.1%)	
Fatigue	Any grade			4 / 4 (66.7%)	4 / 4 (36.4%)	1 / 1 (25.0%)
	Grade 1			4 / 4 (66.7%)	4 / 4 (36.4%)	1 / 1 (25.0%)
**Infections and infestations**				1 / 1 (16.7%)	1 / 1 (9.1%)	
Nasopharyngitis	Any grade			1 / 1 (16.7%)	1 / 1 (9.1%)	
	Grade 1			1 / 1 (16.7%)	1 / 1 (9.1%)	
**Investigations**				1 / 1 (16.7%)	1 / 1 (9.1%)	
Blood pressure systolic increased	Any grade			1 / 1 (16.7%)	1 / 1 (9.1%)	
	Grade 2			1 / 1 (16.7%)	1 / 1 (9.1%)	
**Nervous system disorders**				2 / 2 (33.3%)	2 / 2 (18.2%)	

System Organ Class Preferred Term	Grade	DSM265 Cohort 1A (n=5)	Malarone Cohort 1B (n=6)	DSM265 Cohort 2 (n=6)	DSM265 Cohorts 1A and 2 (n=11)	Placebo Cohorts 1A and 2 (n=4)
Headache	Any grade<br>Grade 1			2 / 2 (33.3%)<br>2 / 2 (33.3%)	2 / 2 (18.2%)<br>2 / 2 (18.2%)	
**Psychiatric disorders**		1 / 1 (20.0%)		1 / 1 (16.7%)	2 / 2 (18.2%)	
Abnormal dreams	Any grade<br>Grade 1			1 / 1 (16.7%)<br>1 / 1 (16.7%)	1 / 1 (9.1%)<br>1 / 1 (9.1%)	
Sleep disorder	Any grade<br>Grade 1	1 / 1 (20.0%)<br>1 / 1 (20.0%)			1 / 1 (9.1%)<br>1 / 1 (9.1%)	
**Respiratory, thoracic and mediastinal disorders**						**1 / 1 (25.0%)**
Cough	Any grade<br>Grade 1					1 / 1 (25.0%)<br>1 / 1 (25.0%)

From PfSPZ challenge up to 1st dose of rescue medication

System Organ Class Preferred Term	Grade	DSM265 Cohort 1A (n=5)	Malarone Cohort 1B (n=6)	DSM265 Cohort 2 (n=6)	DSM265 Cohorts 1A and 2 (n=11)	Placebo Cohorts 1A and 2 (n=4)
**Subjects with any unrelated TEAE**	Any grade	8 / 2 (40.0%)	15 / 5 (83.3%)	10 / 5 (83.3%)	18 / 7 (63.6%)	9 / 4 (100.0%)
	Grade 1<br>Grade 2	8 / 2 (40.0%)	14 / 5 (83.3%)<br>1 / 1 (16.7%)	10 / 5 (83.3%)	18 / 7 (63.6%)	9 / 4 (100.0%)
**Blood and lymphatic system disorders**						2 / 1 (25.0%)
Lymphadenopathy	Any grade<br>Grade 1			1 / 1 (16.7%)<br>1 / 1 (16.7%)	1 / 1 (9.1%)<br>1 / 1 (9.1%)	
**Gastrointestinal disorders**			3 / 1 (16.7%)	2 / 1 (16.7%)	2 / 1 (9.1%)	

System Organ Class Preferred Term	Grade	DSM265 Cohort 1A (n=5)	Malarone Cohort 1B (n=6)	DSM265 Cohort 2 (n=6)	DSM265 Cohorts 1A and 2 (n=11)	Placebo Cohorts 1A and 2 (n=4)
Abdominal pain	Any grade			2 / 1 (16.7%)	2 / 1 (9.1%)	
	Grade 1			2 / 1 (16.7%)	2 / 1 (9.1%)	
Aphthous ulcer	Any grade		1 / 1 (16.7%)			
	Grade 1		1 / 1 (16.7%)			
Constipation	Any grade		2 / 1 (16.7%)			
	Grade 1		2 / 1 (16.7%)			
**General disorders and administration site conditions**		1 / 1 (20.0%)	2 / 2 (33.3%)		1 / 1 (9.1%)	3 / 3 (75.0%)
Chills	Any grade		1 / 1 (16.7%)			
	Grade 1		1 / 1 (16.7%)			
Fatigue	Any grade	1 / 1 (20.0%)	1 / 1 (16.7%)		1 / 1 (9.1%)	3 / 3 (75.0%)
	Grade 1	1 / 1 (20.0%)	1 / 1 (16.7%)		1 / 1 (9.1%)	3 / 3 (75.0%)
**Infections and infestations**		1 / 1 (20.0%)	3 / 3 (50.0%)		1 / 1 (9.1%)	1 / 1 (25.0%)
Nasopharyngitis	Any grade					1 / 1 (25.0%)
Nasopharyngitis	Grade 1					1 / 1 (25.0%)
Upper respiratory tract infection	Any grade		1 / 1 (16.7%)			
	Grade 1		1 / 1 (16.7%)			
Viral upper respiratory tract infection	Any grade	1 / 1 (20.0%)	2 / 2 (33.3%)		1 / 1 (9.1%)	
	Grade 1	1 / 1 (20.0%)	2 / 2 (33.3%)		1 / 1 (9.1%)	
Alanine aminotransferase increased	Any grade			1 / 1 (16.7%)	1 / 1 (9.1%)	
	Grade 1			1 / 1 (16.7%)	1 / 1 (9.1%)	
**Investigations**				1 / 1 (16.7%)	1 / 1 (9.1%)	

System Organ Class Preferred Term	Grade	DSM265 Cohort 1A (n=5)	Malarone Cohort 1B (n=6)	DSM265 Cohort 2 (n=6)	DSM265 Cohorts 1A and 2 (n=11)	Placebo Cohorts 1A and 2 (n=4)
Hypoglycaemia	Any grade		1 / 1 (16.7%)			
	Grade 2		1 / 1 (16.7%)			
**Musculoskeletal and connective tissue disorders**				1 / 1 (16.7%)	1 / 1 (9.1%)	1 / 1 (25.0%)
Myalgia	Any grade			1 / 1 (16.7%)	1 / 1 (9.1%)	1 / 1 (25.0%)
	Grade 1			1 / 1 (16.7%)	1 / 1 (9.1%)	1 / 1 (25.0%)
**Nervous system disorders**		3 / 1 (20.0%)		1 / 1 (16.7%)	4 / 2 (18.2%)	2 / 1 (25.0%)
Dizziness	Any grade	1 / 1 (20.0%)			1 / 1 (9.1%)	
	Grade 1	1 / 1 (20.0%)			1 / 1 (9.1%)	
Headache	Any grade	2 / 1 (20.0%)		1 / 1 (16.7%)	3 / 2 (18.2%)	2 / 1 (25.0%)
	Grade 1	2 / 1 (20.0%)		1 / 1 (16.7%)	3 / 2 (18.2%)	2 / 1 (25.0%)
**Psychiatric disorders**		1 / 1 (20.0%)			1 / 1 (9.1%)	1 / 1 (25.0%)
Insomnia	Any grade					1 / 1 (25.0%)
	Grade 1					1 / 1 (25.0%)
Sleep disorder	Any grade	1 / 1 (20.0%)			1 / 1 (9.1%)	
	Grade 1	1 / 1 (20.0%)			1 / 1 (9.1%)	
**Respiratory, thoracic and mediastinal disorders**		2 / 1 (20.0%)	4 / 2 (33.3%)	4 / 2 (33.3%)	6 / 3 (27.3%)	
Cough	Any grade	1 / 1 (20.0%)		1 / 1 (16.7%)	2 / 2 (18.2%)	
	Grade 1	1 / 1 (20.0%)		1 / 1 (16.7%)	2 / 2 (18.2%)	
Dysphonia	Any grade		1 / 1 (16.7%)	1 / 1 (16.7%)	1 / 1 (9.1%)	
	Grade 1		1 / 1 (16.7%)	1 / 1 (16.7%)	1 / 1 (9.1%)	
Nasal congestion	Any grade		1 / 1 (16.7%)	1 / 1 (16.7%)	1 / 1 (9.1%)	
	Grade 1		1 / 1 (16.7%)	1 / 1 (16.7%)	1 / 1 (9.1%)	

System Organ Class Preferred Term	Grade	DSM265 Cohort 1A (n=5)	Malarone Cohort 1B (n=6)	DSM265 Cohort 2 (n=6)	DSM265 Cohorts 1A and 2 (n=11)	Placebo Cohorts 1A and 2 (n=4)
Oropharyngeal pain	Any grade	1 / 1 (20.0%)	2 / 2 (33.3%)	1 / 1 (16.7%)	2 / 2 (18.2%)	
	Grade 1	1 / 1 (20.0%)	2 / 2 (33.3%)	1 / 1 (16.7%)	2 / 2 (18.2%)	
**Skin and subcutaneous tissue disorders**			2 / 2 (33.3%)			1 / 1 (25.0%)
Hyperhidrosis	Any grade		1 / 1 (16.7%)			
	Grade 1		1 / 1 (16.7%)			
Night sweats	Any grade					1 / 1 (25.0%)
	Grade 1					1 / 1 (25.0%)
Urticaria	Any grade		1 / 1 (16.7%)			
	Grade 1		1 / 1 (16.7%)			

From 1st dose of rescue medication up to End of Study Visit (Day 60)

System Organ Class Preferred Term	Grade	DSM265 Cohort 1A (n=5)	Malarone Cohort 1B (n=6)	DSM265 Cohort 2 (n=6)	DSM265 Cohorts 1A and 2 (n=11)	Placebo Cohorts 1A and 2 (n=4)
**Subjects with any un-related TEAE**	Any grade	10 / 3 (60.0%)	1 / 1 (16.7%)	56 / 6 (100.0%)	66 / 9 (81.8%)	37 / 3 (75.0%)
	Grade 1	7 / 3 (60.0%)	1 / 1 (16.7%)	37 / 6 (100.0%)	44 / 9 (81.8%)	28 / 3 (75.0%)
	Grade 2			12 / 4 (66.7%)	12 / 4 (36.4%)	8 / 3 (75.0%)
	Grade 3	1 / 1 (20.0%)		5 / 3 (50.0%)		
	Grade 4	2 / 1 (20.0%)		2 / 1 (16.7%)	4 / 2 (18.2%)	
**Blood and lymphatic system disorders**				7 / 2 (33.3%)	7 / 2 (18.2%)	1 / 1 (25.0%)
Haemolysis	Any grade			1 / 1 (16.7%)	1 / 1 (9.1%)	
	Grade 1			1 / 1 (16.7%)	1 / 1 (9.1%)	
Leukopenia	Any grade			1 / 1 (16.7%)	1 / 1 (9.1%)	1 / 1 (25.0%)
	Grade 1			1 / 1 (16.7%)	1 / 1 (9.1%)	
	Grade 2					1 / 1 (25.0%)
Lymphopenia	Any grade			3 / 2 (33.3%)	3 / 2 (18.2%)	
	Grade 1			1 / 1 (16.7%)	1 / 1 (9.1%)	
	Grade 2			1 / 1 (16.7%)	1 / 1 (9.1%)	
	Grade 4			1 / 1 (16.7%)	1 / 1 (9.1%)	
Neutropenia	Any grade			1 / 1 (16.7%)	1 / 1 (9.1%)	
	Grade 1			1 / 1 (16.7%)	1 / 1 (9.1%)	
Thrombocytopenia	Any grade			1 / 1 (16.7%)	1 / 1 (9.1%)	
	Grade 1			1 / 1 (16.7%)	1 / 1 (9.1%)	
**Cardiac disorders**		2 / 1 (20.0%)		4 / 3 (50.0%)	6 / 4 (36.4%)	3 / 2 (50.0%)

System Organ Class<br>Preferred Term	Grade	DSM265 Cohort 1A (n=5)	Malarone Cohort 1B (n=6)	DSM265 Cohort 2 (n=6)	DSM265 Cohorts 1A and 2 (n=11)	Placebo Cohorts 1A and 2 (n=4)
Palpitations	Any grade	1 / 1 (20.0%)			1 / 1 (9.1%)	1 / 1 (25.0%)
	Grade 1	1 / 1 (20.0%)			1 / 1 (9.1%)	1 / 1 (25.0%)
Sinus tachycardia	Any grade	1 / 1 (20.0%)			1 / 1 (9.1%)	
	Grade 1	1 / 1 (20.0%)			1 / 1 (9.1%)	
Tachycardia	Any grade			4 / 3 (50.0%)	4 / 3 (27.3%)	2 / 2 (50.0%)
	Grade 1			4 / 3 (50.0%)	4 / 3 (27.3%)	2 / 2 (50.0%)
**Ear and labyrinth disorders**						1 / 1 (25.0%)
Ear pain	Any grade					1 / 1 (25.0%)
			Grade 1			
Eye disorders				1 / 1 (16.7%)	1 / 1 (9.1%)	
Eye pain	Any grade			1 / 1 (16.7%)	1 / 1 (9.1%)	
	Grade 1			1 / 1 (16.7%)	1 / 1 (9.1%)	
**Gastrointestinal disorders**				2 / 2 (33.3%)	2 / 2 (18.2%)	1 / 1 (25.0%)
Nausea	Any grade			1 / 1 (16.7%)	1 / 1 (9.1%)	1 / 1 (25.0%)
	Grade 1			1 / 1 (16.7%)	1 / 1 (9.1%)	1 / 1 (25.0%)
Vomiting	Any grade			1 / 1 (16.7%)	1 / 1 (9.1%)	
	Grade 1			1 / 1 (16.7%)	1 / 1 (9.1%)	
**General disorders and administration site conditions**				16 / 5 (83.3%)	16 / 5 (45.5%)	14 / 3 (75.0%)
Chest discomfort	Any grade					1 / 1 (25.0%)
	Grade 1					1 / 1 (25.0%)

System Organ Class Preferred Term	Grade	DSM265 Cohort 1A (n=5)	Malarone Cohort 1B (n=6)	DSM265 Cohort 2 (n=6)	DSM265 Cohorts 1A and 2 (n=11)	Placebo Cohorts 1A and 2 (n=4)
Chills	Any grade			2 / 2 (33.3%)	2 / 2 (18.2%)	2 / 2 (50.0%)
	Grade 1			2 / 2 (33.3%)	2 / 2 (18.2%)	2 / 2 (50.0%)
Fatigue	Any grade			6 / 5 (83.3%)	6 / 5 (45.5%)	5 / 3 (75.0%)
	Grade 1			4 / 4 (66.7%)	4 / 4 (36.4%)	3 / 3 (75.0%)
	Grade 2			2 / 2 (33.3%)	2 / 2 (18.2%)	1 / 1 (25.0%)
	Grade 3					1 / 1 (25.0%)
Pyrexia	Any grade			8 / 3 (50.0%)	8 / 3 (27.3%)	6 / 2 (50.0%)
	Grade 1			1 / 1 (16.7%)	1 / 1 (9.1%)	3 / 2 (50.0%)
	Grade 2			3 / 3 (50.0%)	3 / 3 (27.3%)	3 / 2 (50.0%)
	Grade 3			4 / 2 (33.3%)	4 / 2 (18.2%)	
**Hepatobiliary disorders**				1 / 1 (16.7%)	1 / 1 (9.1%)	
Hyperbilirubinemia	Any grade			1 / 1 (16.7%)	1 / 1 (9.1%)	
	Grade 1			1 / 1 (16.7%)	1 / 1 (9.1%)	
**Infections and infestations**		1 / 1 (20.0%)	1 / 1 (16.7%)	2 / 2 (33.3%)	3 / 3 (27.3%)	2 / 1 (25.0%)
Rhinitis	Any grade	1 / 1 (20.0%)			1 / 1 (9.1%)	
	Grade 1	1 / 1 (20.0%)			1 / 1 (9.1%)	
Sinusitis	Any grade			2 / 2 (33.3%)	2 / 2 (18.2%)	1 / 1 (25.0%)
	Grade 1			2 / 2 (33.3%)	2 / 2 (18.2%)	1 / 1 (25.0%)
Tonsillitis	Any grade					1 / 1 (25.0%)
	Grade 1					1 / 1 (25.0%)
**Upper respiratory tract infection**	Any grade		1 / 1 (16.7%)			

System Organ Class Preferred Term	Grade	DSM265 Cohort 1A (n=5)	Malarone Cohort 1B (n=6)	DSM265 Cohort 2 (n=6)	DSM265 Cohorts 1A and 2 (n=11)	Placebo Cohorts 1A and 2 (n=4)
	Grade 1		1 / 1 (16.7%)			
**Investigations**		1 / 1 (20.0%)		3 / 1 (16.7%)	4 / 2 (18.2%)	
Alanine aminotransferase increased	Any grade			1 / 1 (16.7%)	1 / 1 (9.1%)	
	Grade 2			1 / 1 (16.7%)	1 / 1 (9.1%)	
Aspartate aminotransferase increased	Any grade			2 / 1 (16.7%)	2 / 1 (9.1%)	
	Grade 1			1 / 1 (16.7%)	1 / 1 (9.1%)	
	Grade 2			1 / 1 (16.7%)	1 / 1 (9.1%)	
Cardiac murmur	Any grade	1 / 1 (20.0%)			1 / 1 (9.1%)	
	Grade 1	1 / 1 (20.0%)			1 / 1 (9.1%)	
**Metabolism and nutrition disorders**				2 / 2 (33.3%)	2 / 2 (18.2%)	
Hyperkalaemia	Any grade			2 / 2 (33.3%)	2 / 2 (18.2%)	
	Grade 3			1 / 1 (16.7%)	1 / 1 (9.1%)	
	Grade 4			1 / 1 (16.7%)	1 / 1 (9.1%)	
**Musculoskeletal and connective tissue disorders**		1 / 1 (20.0%)		4 / 3 (50.0%)	5 / 4 (36.4%)	3 / 2 (50.0%)
Flank pain	Any grade					1 / 1 (25.0%)
	Grade 1					1 / 1 (25.0%)
Myalgia	Any grade	1 / 1 (20.0%)		4 / 3 (50.0%)	5 / 4 (36.4%)	2 / 2 (50.0%)
	Grade 1	1 / 1 (20.0%)		3 / 3 (50.0%)	4 / 4 (36.4%)	2 / 2 (50.0%)
	Grade 2			1 / 1 (16.7%)	1 / 1 (9.1%)	
**Nervous system disorders**		1 / 1 (20.0%)		8 / 5 (83.3%)	9 / 6 (54.5%)	10 / 3 (75.0%)

System Organ Class Preferred Term	Grade	DSM265 Cohort 1A (n=5)	Malarone Cohort 1B (n=6)	DSM265 Cohort 2 (n=6)	DSM265 Cohorts 1A and 2 (n=11)	Placebo Cohorts 1A and 2 (n=4)
Dizziness	Any grade					2 / 2 (50.0%)
	Grade 1					2 / 2 (50.0%)
Headache	Any grade			8 / 5 (83.3%)	8 / 5 (45.5%)	8 / 3 (75.0%)
	Grade 1			5 / 4 (66.7%)	5 / 4 (36.4%)	5 / 2 (50.0%)
	Grade 2			3 / 3 (50.0%)	3 / 3 (27.3%)	3 / 3 (75.0%)
Loss of consciousness	Any grade	1 / 1 (20.0%)			1 / 1 (9.1%)	
	Grade 4	1 / 1 (20.0%)			1 / 1 (9.1%)	
**Respiratory, thoracic and mediastinal disorders**		2 / 1 (20.0%)		2 / 1 (16.7%)	4 / 2 (18.2%)	1 / 1 (25.0%)
Cough	Any grade			1 / 1 (16.7%)	1 / 1 (9.1%)	
	Grade 1			1 / 1 (16.7%)	1 / 1 (9.1%)	
Dyspnoea	Any grade	1 / 1 (20.0%)		1 / 1 (16.7%)	2 / 2 (18.2%)	1 / 1 (25.0%)
	Grade 1	1 / 1 (20.0%)		1 / 1 (16.7%)	2 / 2 (18.2%)	1 / 1 (25.0%)
Pulmonary embolism	Any grade	1 / 1 (20.0%)			1 / 1 (9.1%)	
	Grade 4	1 / 1 (20.0%)			1 / 1 (9.1%)	
**Skin and subcutaneous tissue disorders**		1 / 1 (20.0%)		3 / 3 (50.0%)	4 / 4 (36.4%)	1 / 1 (25.0%)
Acne	Any grade	1 / 1 (20.0%)			1 / 1 (9.1%)	
	Grade 1	1 / 1 (20.0%)			1 / 1 (9.1%)	
Hyperhidrosis	Any grade			3 / 3 (50.0%)	3 / 3 (27.3%)	1 / 1 (25.0%)
	Grade 1			3 / 3 (50.0%)	3 / 3 (27.3%)	1 / 1 (25.0%)
**Vascular disorders**		1 / 1 (20.0%)		1 / 1 (16.7%)	2 / 2 (18.2%)	

System Organ Class Preferred Term	Grade	DSM265 Cohort 1A (n=5)	Malarone Cohort 1B (n=6)	DSM265 Cohort 2 (n=6)	DSM265 Cohorts 1A and 2 (n=11)	Placebo Cohorts 1A and 2 (n=4)
Deep vein thrombosis	Any grade	1 / 1 (20.0%)			1 / 1 (9.1%)	
	Grade 3	1 / 1 (20.0%)			1 / 1 (9.1%)	
Hypertension	Any grade			1 / 1 (16.7%)	1 / 1 (9.1%)	
	Grade 1			1 / 1 (16.7%)	1 / 1 (9.1%)	
